# Multi‐Energy‐Driven Photocatalysis: Mechanism, Progress, and Perspective

**DOI:** 10.1002/EXP.20240195

**Published:** 2026-05-26

**Authors:** Liangcheng Xu, Xinrong Zhang, Duan Yu, Yingjuan Zhang, Jung‐Ho Yun, Songcan Wang

**Affiliations:** ^1^ State Key Laboratory of Flexible Electronics & Institute of Flexible Electronics Northwestern Polytechnical University Xi'an China; ^2^ Queen Mary University of London Engineering School Northwestern Polytechnical University Xi'an China; ^3^ Air & Environment Energy Nexus (A2EN) Lab, Department of Environmental Science and Engineering, College of Engineering Kyung Hee University Gyeonggi‐do Republic of Korea

**Keywords:** electrical energy, magnetic energy, mechanical energy, microwave energy, multi‐energy field, photocatalysis, thermal energy

## Abstract

Photocatalysis, a technology that can convert solar energy into chemical energy, exhibits tremendous potential for addressing the current environmental pollution and energy crisis. In the past decades, a variety of regulating strategies, including element doping, defect construction, and band engineering, have been developed to improve the performance of photocatalysis. However, single‐mode photocatalysis has reached its performance limitation. To further enhance the photocatalytic performance, an emerging strategy is to construct multi‐energy‐integrated photocatalysis. This strategy has been proven to broaden the light absorption range and enhance the charge separation efficiency. In this review, different types of external energy, including thermal energy, electrical energy, magnetic energy, mechanical energy, and microwave energy for enhancing the performance of photocatalysis, are classified. The fundamental reinforcement mechanisms and advantages of external energy–driven photocatalysis are critically discussed. The state‐of‐the‐art progress of the utilization of external fields in photocatalytic water splitting, pollutant degradation, and chemical synthesis is summarized. Finally, the challenges and future prospects of this promising field are demonstrated.

## Graphical abstract

Photocatalysis technology can be utilized in water splitting, carbon dioxide reduction, pollutant degradation, and chemical synthesis. Introducing external energy fields, including thermal energy, electrical energy, magnetic energy, mechanical energy, and microwave energy, has been considered as a promising method to improve photocatalytic performance in recent years. This article focuses on fundamental mechanisms and advantages of the multi‐energy integrated photocatalysis process, providing deeper insights into designing highly efficient photocatalytic systems.

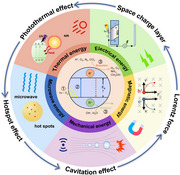

## 1 Introduction

Since excessive emissions of greenhouse gases lead to climate change and environmental crises, carbon neutrality as a global goal has received a positive response in recent years [1]. It is crucial to limit global warming to 1.5°C or 2°C above pre‐industrial levels, as outlined in the Paris Agreement [2]. Consequently, replacing conventional fossil fuels through harnessing renewable energy such as solar, wind, and geothermal energy, or applying fuel cells to convert the chemical energy stored in fuels to electricity, is a promising strategy to solve the climatic and environmental issues [3, 4, 5]. Among these renewable energies, solar energy has been considered as a vital alternative to conventional fossil energy due to its clean, safe, and sustainable features. Converting solar energy at low cost and with high efficiency is of great importance. It is particularly transformative for remote or underserved areas, providing electricity and clean fuels where grid infrastructure is absent [6, 7]. Photocatalysis is a direct solar energy conversion technology, which can directly transform the incident photons into valuable chemicals [8, 9, 10, 11]. At present, photocatalysis technology has been applied in the field of water splitting, carbon dioxide (CO_2_) reduction, organic pollutant degradation, as well as organic chemical synthesis [12, 13, 14, 15, 16].

The photocatalytic process is composed of three crucial steps: (1) generation of photogenerated electron–hole pairs by solar energy absorption, (2) charge separation and transport in the bulk and at the surface of a semiconductor photocatalyst, (3) redox reaction at the photocatalyst surface. The ultimate solar energy conversion efficiency depends on the separation efficiency of each step. To significantly improve the solar energy conversion efficiency of these three fundamental steps, considerable effects have been made [17]. Owing to the developments of new materials, the light absorption range of semiconducting materials has been extended to visible light and even the full spectrum, such as Ta_3_N_5_ [18], SrTiO_3_ [19], and perovskite‐based materials [20, 21, 22]. Emerging materials exhibit a higher theoretical solar energy conversion efficiency due to their suitable band structure and unique physical and chemical properties compared with the conventional photocatalysts, including metal oxides (e.g., TiO_2_, Fe_2_O_3_, WO_3_, Cu_2_O, BiOI, BiVO_4_) [23, 24, 25] and carbon‐based materials (e.g., g‐C_3_N_4_, GO, rGO) [26, 27, 28]. In addition, energy band engineering strategies, including element doping and heterojunction construction, are utilized in conventional photocatalytic semiconductors and show excellent improvement in broadening the light absorption range [29, 30].

Effective charge separation and transport in photocatalysis play a vital role in improving the whole photocatalytic efficiency [31]. Previous research has confirmed that heterogeneous element doping [32], defect construction [33], and crystal facet engineering [34] are effective strategies for restraining the recombination of photoexcited electrons and holes in bulk semiconductors [35, 36, 37, 38]. In particular, an increasing number of research works focused on defect engineering, such as the generation of oxygen vacancies, metal atom vacancies, and sulfur vacancies in lattices [39, 40]. These vacancy defects can create localized electronic states within the bandgap, where charge carriers are trapped by these states, leading to an effective spatial separation of the photogenerated electron–hole pairs. For example, an optimized number of vacancy defects in BiVO_4_ photocatalysts can improve the electronic conductivity and charge separation efficiency. As a result, a much higher photocatalytic performance can be observed in water splitting [41]. Moreover, defect engineering on the active sites of the semiconducting surface would significantly facilitate the adsorption of reactants or desorption of products, thus enhancing surface reaction kinetics. However, single‐mode photocatalysis cannot fully utilize solar energy and is limited by an inadequate kinetic response and suppression of charge transfer. Additionally, current regulation strategies for light absorption, charge separation, and reaction kinetics generally cause changes in morphology, composition, and crystal structure by complex preparation methods. It is difficult to improve the single energy band alignment without introducing other drawbacks, such as carrier recombination centers and crystal deformation. Therefore, the introduction of other energy sources into photocatalysis to form multi‐energy integrated photocatalysis systems has attracted considerable attention in recent years. For example, a Cs_3_Sb_2_I_9_ photocatalyst was developed to reduce CO_2_ to CH_4_ and CO at a high efficiency by synergistic photothermal catalysis and exhibited a higher production rate of 95.7 µmol g^−1^ h^−1^ without any sacrificial agents compared to the single‐mode photocatalysis system counterparts (1.1 µmol g^−1^ h^−1^), implying a tremendous potential of multi‐energy integrated photocatalysis systems in the field of CO_2_ reduction [42].

Besides the excitation energy of solar light, other external energy, composed of thermal energy, electrical energy, magnetic energy, mechanical energy, and microwave (MW) energy, can also be applied to photocatalysis [43]. Since the introduction of other energies usually relies on external fields, the effects of multiple energies on the physical–chemical properties of semiconductors are negligible. In the presence of external energy, the light absorption range, charge carrier separation, surface reaction kinetics, and selectivity for desired chemicals can be improved in comparison to single‐mode photocatalysis [43]. The fundamental mechanisms of different external energies for improving the photocatalytic performance are significantly different. Thermal and MW energy can heat semiconductor materials with overall heating and local heating, respectively. Elevated temperatures effectively enhance the mobility of charge carriers, thus leading to the restricted recombination of electron–hole pairs. Electric and mechanical energy can generate a charge separation field, called the built‐in electric field and the built‐in polarizing electric field, respectively. Benefiting from the charge separation field, the recombination of electrons and holes can be significantly inhibited. Lorentz force and changed spin state induced by magnetic energy also have a strong power to drive the separation of photogenerated electrons and holes [44]. Considering the electrical energy as an example, an external electric field creates a space charge layer (SCL) at the photoelectrode/electrolyte interface due to the change of the Fermi level, where the band bending effectively improves the separation and transfer of the photogenerated electrons and holes [45]. Based on the important role of an externally applied bias for separating charges and inhibiting recombination, a photoelectrochemical (PEC) system was established and exhibited tremendous potential in the area of water splitting and chemical synthesis. The latest research results show that a perovskite‐based PEC system can simultaneously conduct ammonia (NH_3_) production and glycerol oxidation at the cathode and anode, respectively. Amazingly, due to the high photovoltaic voltage and narrow bandgap of perovskite‐based semiconductors, the entire PEC device is bias‐free without the input of external electric energy. The photocurrent density of the PEC NH_3_ production system coupled to the glycerol oxidation device reaches a photocurrent density of 21.2 mA cm^−2^ without any applied voltage, which is obviously higher than the theoretical photocurrent density of WO_3_ (4.8 mA cm^−2^), BiVO_4_ (7.5 mA cm^−2^), and Fe_2_O_3_ (12.6 mA cm^−2^) with a bias of 1.23 V vs. reversible hydrogen electrode (RHE) [46].

In addition, magnetic‐field‐induced Lorentz force can also provide enough driving forces for separating the photoexcited electrons and holes, thus enhancing the photocatalytic efficiency. Essentially, the separation of charge carriers originates from the oppositely moving direction of electrons (negative charge) and holes (positive charge), which then react with reactants to participate in redox reactions [47]. The external Lorentz force could induce the spatial separation of electrons and holes because charges with opposite electronic properties have opposite migration directions. As an emerging route, the utilization of thermal‐assisted photocatalysis has received increasing attention recently. Some metals, such as Au, Pt, and their alloy particles that can respond to infrared (IR) light, were successfully loaded on the photocatalyst surface. Their localized surface plasmon resonance (LSPR) effect upon the irradiation of solar energy enables electrons to enrich and absorb photons in a space far larger than the geometric cross section, thus improving photocatalytic performance [48]. Owing to the significant improvement of external energy input on light absorption, charge separation, and reaction selectivity in photocatalysis, a large number of reviews about single‐energy field coupled photocatalysis have been published. Li and coworkers summarized the recent progress of magnetic field‐coupled photocatalysis, revealing the effects of magnetoresistance (MR), Lorentz force, and spin polarization on crucial processes in photocatalysis [49]. Fang and coworkers first defined thermo‐assisted photocatalysis, thermo‐photo cocatalysis, and photo‐promoted thermal catalysis, summarizing various thermal‐field‐coupled photocatalytic mechanisms. However, little attention has been paid to multi‐energy integrated photocatalysis [48]. Moreover, critical analysis and discussion of the basic mechanisms of the multi‐energy field for enhancing photocatalytic performance are necessary.

**FIGURE 1:**
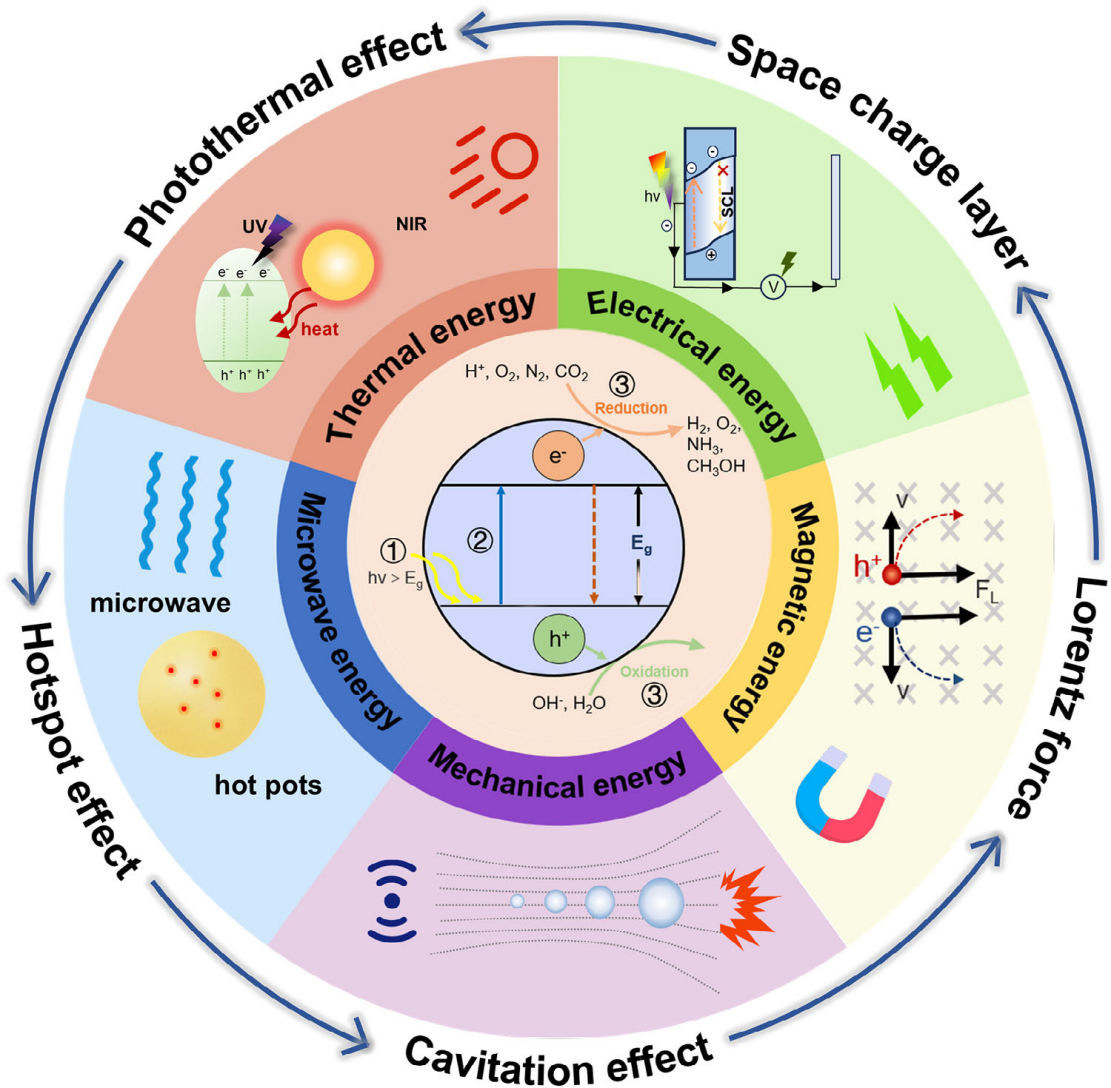
Schematic of the multi‐energy integrated photocatalysis.

In this review, we critically analyze and discuss recent progress in the application of different types of energy fields in photocatalysis, comprising thermal, electrical, magnetic, mechanical, and MW energy. This multi‐energy integrated photocatalysis shows immense potential for practical photocatalysis applications, such as clean energy production, CO_2_ reduction, organic pollutants degradation, valuable chemical synthesis, and artificial nitrogen fixation. In addition, an in‐depth investigation into fundamental mechanisms underlying the influence of the multi‐energy field on crucial photocatalytic steps is highlighted, as shown in Figure 1. Finally, the future prospects for developing multi‐energy integrated photocatalysis are presented.

## 2 Mechanisms and Limitations of Conventional Photocatalysis

### 2.1 Working Principle of Photocatalysis

**FIGURE 2:**
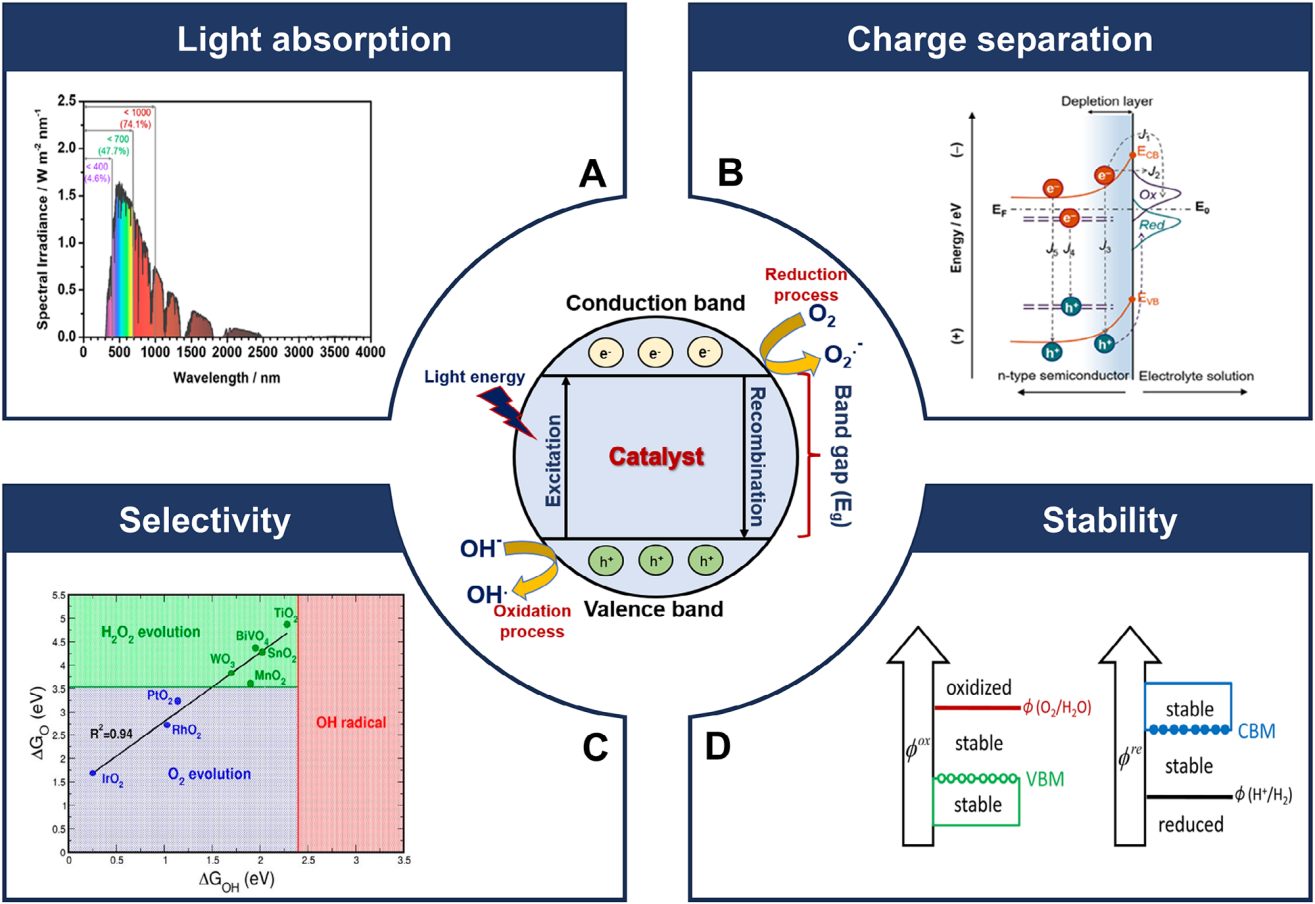
Schematic illustration of the working principles and challenges of conventional photocatalysis. (A) AM 1.5 G solar spectrum on the basis of the ASTM G173‐03 reference spectrum [60]. Copyright 2020, Royal Society Chemistry. (B) Electron energy diagram of an n‐type semiconductor and different charge recombination pathways by shallow energy level or surface state [60]. Copyright 2020, Royal Society Chemistry. (C) Phase diagram based on the binding energies of O* versus OH* [75]. Copyright 2017, American Chemical Society. (D) Schematic diagram of stability variation for photoelectrodes with the change of semiconductor redox potentials (ϕ^ox^ and ϕ^re^) [81]. Copyright 2012, American Chemical Society.

Photocatalytic reactions are heavily dependent on the wavelength of solar light and the band structure of semiconductor photocatalysts. The fundamental photocatalytic process can be classified into three steps: (1) light absorption; (2) photogenerated carrier transfer and separation; and (3) surface chemical reactions (i.e., oxidation semi‐reaction and reduction semi‐reaction) [50, 51]. As shown in Figure 2, when a semiconductor is irradiated by the light energy in terms of photons, electrons in the valence band (VB) will be excited to the conduction band (CB) if the energy of the incident light is greater than the bandgap energy of the semiconductor materials. Owing to the transition of electrons from the VB to the CB, holes would be left in the VB. In an ideal situation, all the excited holes and electrons are transferred to the oxidation sites and reduction sites on semiconductor surfaces to participate in the oxidation semi‐reaction and reduction semi‐reaction, respectively. However, large amounts of electron–hole pairs undergo recombination, thus reducing the photocatalytic efficiency. The recombination of electrons and holes is mainly divided into bulk recombination and surface recombination [52]. Before photoexcited carriers reach the surface, defects in the crystal lattice trap charge carriers, leading to serious recombination. In addition, the poor charge mobility of semiconductors is also a reason for recombination. For instance, Fe_2_O_3_ as a semiconductor can absorb a wavelength of lower than 590 nm in a solar spectrum, obviously greater than rutile TiO_2_ (≤413 nm) and BiVO_4_ (≤520 nm) [53]. Nevertheless, the photocatalytic performance of rutile TiO_2_ and BiVO_4_ is significantly higher than that of Fe_2_O_3_. This abnormal phenomenon originates from different hole mobility, where the hole mobility of Fe_2_O_3_ (0.0001 cm^2^ V^−1^ s^−1^) is remarkably lower than that of BiVO_4_ (0.02–0.044 cm^2^ V^−1^ s^−1^) and rutile TiO_2_ (1 × 10^−4^–1 × 10^−3^ cm^2^ V^−1^ s^−1^) [54]. If the photogenerated electrons and holes reach the surface simultaneously and do not find suitable reactants, the electrons and holes tend to recombine quickly, either radiatively (emitting light) or non‐radiatively (releasing heat). The whole solar energy conversion efficiency (*η*_total_) can be calculated through the equation [54]

(1) $$\;\eta_{\mathrm{total}}=\eta_{\mathrm{absorption}}\;\times \eta_{\mathrm{separation}}\times \eta_{\mathrm{reaction}}$$

where *η*_absorption_ represents the fraction of photogenerated carriers excited by incident solar energy, *η*_separation_ is defined as the fraction of charges reaching the semiconductor/solution interface, and *η*_separation_ is the reaction efficiency.

### 2.2 Current Challenges of Photocatalysis

Although practical applications of photocatalysis have made tremendous progress in the field of water splitting, pollutant degradation, and CO_2_ reduction during the last three decades, photocatalysis still faces a variety of challenges, which can be summarized as four aspects according to the fundamental photocatalytic steps [55]. The first challenge is limited light absorption. A number of photocatalysts (e.g., TiO_2_) primarily absorb ultraviolet (UV) light, which occupies only 4% of the solar spectrum, seriously wasting the visible light (45% of the solar spectrum) [56]. The second challenge is the charge separation. Photogenerated charge carriers tend to recombine rapidly (nanosecond timescales), reducing their availability for complex redox reactions [57]. The third challenge is selectivity and reaction kinetics. Common photocatalysis reactions, including CO_2_ reduction or water splitting, often generate mixed products (e.g., CO, CH_4_, HCOOH in CO_2_ reduction) and form undesired side reactions (e.g., Cl^−^ oxidation in seawater splitting), which reduce the productivity of the target products [58]. The fourth challenge is material stability and durability. A variety of semiconductors (e.g., Cu_2_O and CdS) can be degraded under extended irradiation or harsh reaction conditions such as acidic/alkaline electrolytes. The instability of semiconductors in actual operation limits their further commercial applications [59]. In this section, we will introduce and discuss the above‐mentioned four main challenges of photocatalysis and briefly summarize the relevant strategies.

#### 2.2.1 Limited Light Absorption

The sun emits electromagnetic waves ranging from X‐rays to radio waves, but the most intense solar energy exists in the visible light range. As represented in Figure 2A, the spectral wavelength from 400 to 1000 nm occupies an overall spectral irradiation energy of approximately 70%. Furthermore, 43% of the solar energy is visible light with a wavelength ranging from 400 to 700 nm [60]. Semiconductor photoabsorbers can absorb solar energy in the form of incident photons and convert them into charge carriers (i.e., photogenerated electrons and holes). The conversion efficiency from incident photons to charge carriers is defined as the quantum efficiency (QE), which is a crucial parameter that describes the device's sensitivity to light and can be calculated by the following equation [61]:

(2) $$\mathrm{QE}\;\left(\lambda \right)=\frac{\mathrm{number}\;\mathrm{of}\;\mathrm{electrons}\;\mathrm{or}\;\mathrm{hole}\;\mathrm{collected}}{\mathrm{number}\;\mathrm{of}\;\mathrm{incident}\;\mathrm{photons}}\;$$

Assuming that semiconductors can absorb the solar energy with 100% of QE, the rutile TiO_2_ can only absorb the UV light up to 400 nm, corresponding to a solar‐to‐hydrogen (STH) efficiency of about 2% [62]. Broadening the light absorption wavelength up to 700 and 1000 nm could significantly increase the theoretical STH to 25% and 47%, which satisfies the commercial requirements for solar hydrogen production (STH>10%) [63]. Therefore, to achieve a highly efficient photocatalytic system, it is crucial to focus on extending the light absorption wavelength to the visible light region or even the near‐IR region. However, large‐scale hydrogen production using photocatalytic technology is still a challenge. Current photocatalytic performance is usually measured in a small‐scale photocatalysis device, which can obtain a higher photocatalytic efficiency than a large‐scale photocatalysis system. For instance, Domen and coworkers designed a 100 m^2^ array of panel reactors to produce hydrogen, reaching a STH of 0.76% [64]. It can be found that the photocatalytic efficiency will decrease with the increase of the reaction area, which limits the development of commercial applications. Therefore, a large‐scale hydrogen production apparatus with a higher STH requires further research.

#### 2.2.2 Charge Separation

A kinetic competition between desired catalysis reactions and the recombination of photoexcited electron–hole pairs is the biggest challenge for designing highly efficient photocatalysts and devices, especially compared with solar cells, where the electrons and holes are extracted on a timescale from nanoseconds to microseconds [65, 66, 67]. To promote the electrons and holes reaching the redox‐active sites, enhancing the charge separation and transfer efficiencies has been considered as a promising method in recent years. The primary work is to understand the mechanism of recombination. The recombination process generally occurs via radiative (band‐to‐band) recombination, that is, recombination by defects in crystal lattices [68]. As shown in Figure 2B, some of the photoexcited electrons and holes are transferred to the reduction sites and oxidation sites on a semiconductor surface. Meanwhile, a large portion of electrons and holes recombine through the “shallow” states arising from crystal defects in the forbidden gap [60]. It should be noted that these “shallow” states that are energetically close to the CB (or the VB) can also increase the conductivity of semiconductors, facilitate the formation of built‐in electric fields in the SCL, and enhance visible light absorption [69].

The other bulk charge recombination is the Schottky–Read–Hall recombination process, where photoexcited carriers in bulk are trapped in the intra‐bandgap states, which can mediate the bulk charge recombination. Different semiconductors typically exhibit different bulk recombination kinetics. For instance, anatase TiO_2_ exhibits a bulk recombination rate 100–1000 times slower than hematite (Fe_2_O_3_), which can be attributed to the localized Fe‐centered d‐d nature of the optical transition in hematite [60]. Besides, the extracted electrons and holes from the semiconductor bulk would undergo a surface recombination process due to the different surface reaction kinetics. As an example, the timescale of water oxidation semi‐reaction induced by holes is between milliseconds and seconds, nearly four times the magnitude of the water reduction semi‐reaction induced by electrons. The left holes are trapped in the surface states, leading to a potential drop across the Helmholtz layer and a band edge unpinning at the same time [70, 71]. It further contributes to serious surface charge recombination, resulting in slow charge separation and transport. With the development of new characteristic technologies composed of time‐resolved X‐ray spectroscopy and in situ spectroscopic research, a deeper understanding of the recombination kinetics has been achieved. Current research should focus on designing appropriate compound semiconductor materials based on kinetic studies.

#### 2.2.3 Selectivity and Reaction Kinetics

Photocatalytic reactions, such as water splitting and pollutant removal, end with the consumption of photogenerated charge carriers reaching the surface active sites. Previous research has confirmed that sluggish surface reaction kinetics would cause negative effects on the overall photocatalysis performance [72, 73]. As an example, slower water oxidation kinetics by the photoexcited holes can induce a fast charge recombination due to the serious accumulation of holes on the photocatalyst surface. Moreover, accumulated holes oxidize semiconductors, leading to poor photostability. To systematically investigate the surface kinetics, transient absorption spectroscopy technologies were applied in classic photocatalysis semiconductors, including WO_3_, TiO_2_, and Fe_2_O_3_. Experimental results illustrated that the average timescale to oxidize water molecules in TiO_2_ required 0.3–1 s at the surface. Similar measured results were observed in WO_3_ and Fe_2_O_3_, in which the water oxidation kinetic timescale for WO_3_ and Fe_2_O_3_ was <1 ms and 3 s, respectively [74]. Therefore, the slow timescale for water oxidation is a major challenge in photocatalytic water splitting. In addition to producing oxygen, water molecules can be oxidized to H_2_O_2_ or ·OH radicals through two‐electron and single‐electron reactions. By calculating the absorption free energy of the relevant reaction intermediate in one‐ (·OH radicals), two‐ (H_2_O_2_), and four‐electron (O_2_) water oxidation reactions for different metal oxides, the two‐dimensional selectivity diagram for three products can be obtained according to the binding energies of O* versus OH*, as shown in Figure 2C. The selectivity diagram is divided into three regions: O_2_ evolution (highlighted in the blue region); H_2_O_2_ evolution (highlighted in the green region); and ·OH radicals (highlighted in the red region). It clearly reveals that a higher O* adsorption energy and a higher OH* absorption free energy are beneficial for the production of H_2_O_2_ and ·OH radicals, respectively. TiO_2_, which is close to the border of OH* absorption energy (red region) in H_2_O_2_ evolution (green region), is known to produce H_2_O_2_ and degrade organic pollutants by producing the ·OH radicals [75].

Recently, CO_2_ reduction by photocatalytic technologies received huge attention, but the selectivity for desired products, such as methane (CH_4_), methanol (CH_3_OH), formic acid (HCOOH), and formaldehyde (CH_2_O), remains a tremendous challenge. Previous reports have shown that nanoparticle (NP) size, crystallinity, and crystal plane would result in different selectivity for CO_2_ reduction. For instance, g‐C_3_N_4_/Pt (111) photocatalysts exhibited a higher selectivity toward CH_3_OH in comparison to g‐C_3_N_4_/Pt (100) due to better CO_2_ absorption and CH_3_OH desorption [76]. Similar selectivity challenges can be found in biomass conversion by photocatalysis and photoelectrocatalysis. Specifically, in the process of glycerol oxidation toward value‐added chemicals, realizing C–OH oxidation to C = O oxidation without breaking down other functional groups is a desired chemical reaction, but the C–C bond cleavage and oxidation to carboxyl as a side reaction significantly decreases the selectivity toward the higher value‐added chemicals. Therefore, a deeper understanding of the mechanism of C–C cleavage should be deeply studied [77].

#### 2.2.4 Stability and Durability

The photocatalytic performance has made great progress in water splitting, CO_2_ reduction, biomass oxidation, and pollutant degradation. In the case of the BiVO_4_ photoanode, Yang and coworkers designed a Fe‐N co‐doped BiVO_4_ photoanode (Fe‐N‐BiVO_4_) through an N‐coordinated Fe precursor with a surface catalyst (FeNiOOH), which exhibited a photocurrent density of 7.01 mA/cm^2^ at 1.23 V vs. RHE and a 20 h stability [78]. Since BiVO_4_ as photoanodes can reach a theoretical photocurrent density of approximately 7.5 mA/cm^2^ at 1.23 V vs. RHE according to its light absorption wavelength (<520 mm), it is difficult to further increase the photocurrent density. Aside from bismuth (Bi)‐based semiconductors, the photocurrent density of some metal sulfides has reached a photocurrent density as high as 15.4 mA/cm^2^, which has satisfied the lowest standard of practical commercialization for solar energy hydrogen production (STH > 5%) [79]. Therefore, ensuring long‐term operational stability is an urgent issue that needs to be solved. The failure mechanism of photocatalysts mainly originates from photoinduced corrosion (degradation or decomposition) under illumination [80].

When photocatalysts are in contact with the solution, the photogenerated holes not only can oxidize the reactants but also can oxidize themselves. The photogenerated electrons have a similar result, where they can be utilized to reduce reactants or themselves. Whether photogenerated carriers are used to corrode themselves is dependent on the thermodynamic oxidation potential (ϕ^ox^) and thermodynamic reduction potential (ϕ^re^) of a semiconductor itself, which are intrinsic physical chemistry properties of semiconductors and can be changed through the pH value. From the perspectives of band structure, the redox potentials of photocatalytic water splitting can be divided into the oxygen evolution potential (ϕ(O_2_/H_2_O), 1.23 V vs. RHE) and the hydrogen evolution potential (ϕ(H^+^/H_2_), 0 V vs. RHE). In general, a photocatalytic water splitting reaction can occur when the VB and CB of a semiconductor are higher than the ϕ(O_2_/H_2_O) and lower than the ϕ(H^+^/H_2_), respectively. However, photogenerated holes tend to oxidize themselves instead of oxidizing water molecules if the ϕ^ox^ is higher than the ϕ(O_2_/H_2_O), while photogenerated electrons tend to reduce themselves instead of reducing water molecules if the ϕ^re^ is lower than the ϕ(H^+^/H_2_). Generally speaking, the stability of photocatalysts relies on the ϕ^ox^ relative to the ϕ(O_2_/H_2_O), and the ϕ^re^ relative to the ϕ(H^+^/H_2_), as shown in Figure 2D [81]. It should be noted that the water redox potentials of ϕ(O_2_/H_2_O) and ϕ(H^+^/H_2_) depend on the pH value on the basis of the Nernstian equation, and the semiconductor redox potentials of ϕ^ox^ and ϕ^re^ are determined by the specific reactions. Therefore, regulating the pH values of a solution may be a promising method to inhibit the serious photocorrosion for semiconductor photocatalysts. At present, a universal method to restrict photocorrosion is to improve interfacial charge separation and transport because it can effectively inhibit the accumulation of carriers on the semiconductor surface, especially for metal oxides, including BiVO_4_, WO_3_, and Fe_2_O_3_ [82, 83, 84, 85]. For large‐scale commercialization, current stability still cannot meet the commercial standard compared with silicon‐based solar cells, which can stably operate for over 20 years. Hence, more attention should be focused on device stability.

## 3 Fundamental Mechanisms and Merits of the External‐Energy Photocatalytic Process

The applications of external‐energy fields to effectively improve the photocatalytic performance have been regarded as a convenient and contactless technology. Compared with the conventional methods that focus on the modification of semiconductor materials, external‐energy fields mainly focus on the promoting effects of external energy on the behavior of charge carriers, which can significantly enhance interfacial charge separation and reaction kinetics. Up to now, thermal energy, electrical energy, magnetic energy, mechanical energy, and MW energy have been introduced into photocatalysis and exhibited enhancements in photocatalytic performance from different aspects. In this section, fundamental mechanisms and merits of five energy fields for improving the photocatalytic performance will be clearly summarized and discussed.

### 3.1 Thermal Field Effects on Charge Separation and Selectivity

Thermal energy plays a vital role in thermo‐assisted photocatalysis for improving the photocatalytic activity. Firstly, heating can provide additional energy to overcome the recombination barrier between photogenerated electrons and holes, thereby enhancing the charge separation efficiency. Through three fundamental mechanisms: (1) plasmonic photothermal effect; (2) nonradiative relaxation in excited semiconductors; and (3) thermal vibrations of molecules in a light‐to‐heat conversion process, where the solar energy is successfully converted to thermal energy, thus effectively increasing the temperature of photocatalysts [86]. With an elevated temperature, the mobility of charge carriers increases to a high level, leading to a reduced likelihood of recombination and ensuring more photogenerated electrons and holes participate in redox reactions. Secondly, thermal energy can effectively enhance the mass transfer during photocatalysis, such as diffusion of reactants and products by liquid and gas media, adsorption, and desorption on the photocatalyst surface [87]. The diffusion coefficient in the liquid phase (*D*_L_) is dependent on the temperature and can be calculated by the Stokes‐Einstein equation [48].

(3) $$\;D_{L}=7.4\times 10^{-8}\frac{T{\left(\psi M\right)}^{0.5}}{{\left(\mu V\right)}^{0.6}}$$

in which *T*, *ψ*, *M*, *m*, *μ*, and *V* refer to the temperature, the solute–solvent interaction factor, the molecular weight of solvent, the viscosity of solvent, and the molar volume of solute at a boiling point, respectively. It is obvious that the diffusion coefficient (*D*_L_) increases with elevated temperature.

Similar to the diffusion coefficient in the liquid phase, the gas phase diffusion coefficient (*D*_G_) also increases with the elevated temperature, and it can be calculated through the Chapman–Enskog equation [48].

(4) $$\;D_{G}=1.86\times 10^{-3}\frac{T^{3/2}\sqrt{1/M_{1}+1/M_{2}}}{P\sigma^{2}\Omega }$$

where *T*, *M*_1_, *M*_2_, *P*, *σ*, and *Ω* represent the temperature, the molecular weight of solute gas, the molecular weight of solvent gas, the pressure of the system, the average collision diameter, and the temperature‐dependent collision integral, respectively.

Due to different interfacial friction resistance between the liquid phase and gas phase, the diffusion coefficient can undergo a significant enhancement when the liquid is evaporated into gas at a rising temperature. For instance, the D_L_ of hydrogen in water (100°C) is 5.03 × 10^−5^, while in the steam phase, the D_G_ is 2.65 × 10^−3^. The 50‐time enhancement in diffusion coefficient contributes to the improvement of mass transfer, resulting in an improved photocatalytic performance [88]. Especially for insoluble gaseous reactants, including methane (CH_4_) and carbon dioxide (CO_2_), the stronger mass transfer can easily facilitate the contact of gaseous reactants with the photocatalyst surface.

Thirdly, the introduction of thermal energy into a photocatalytic system is a promising strategy for promoting surface species transfer, which is the key mediator for oxidation and reduction semi‐reactions. Given a case of CO_2_ reduction, protons generated in the oxidation semi‐reaction are transferred to the active reduction sites to participate in the CO_2_ reduction semi‐reaction. The rate‐limiting step during the CO_2_ reduction process is proton transfer instead of electron transfer, which can be attributed to a smaller time scale of hundreds of microseconds for the proton transfer compared with a few picoseconds for the electron transfer [89].

**FIGURE 3:**
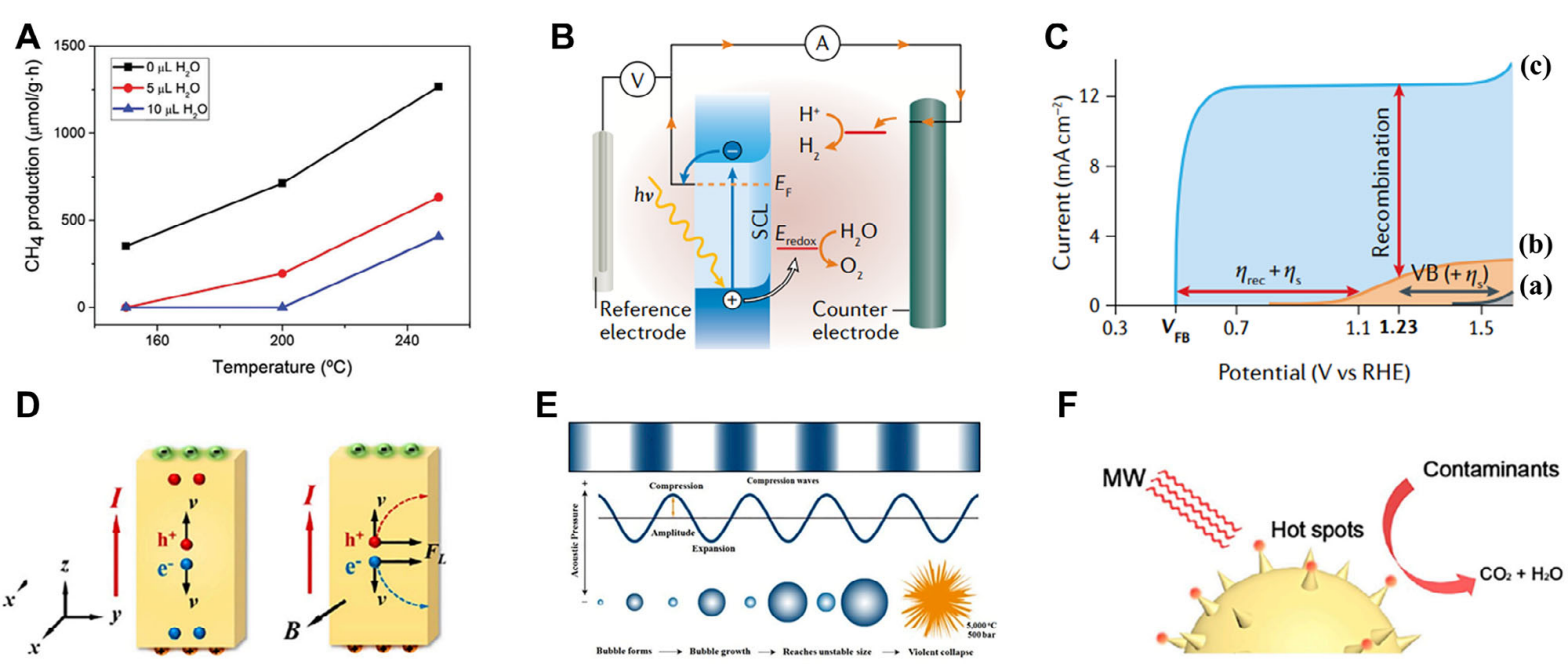
(A) Temperature‐dependent curves of CH_4_ production rate under the addition of trace amounts of H_2_O (0–10 µL) with a light intensity of 200 mW cm^−2^. The reaction time is 1 h [90]. Copyright 2017, Royal Society Chemistry. (B) Schematic diagram of PEC water splitting, in which the space charge layer creates a band bending region that can promote photogenerated electron–hole pairs transport and separation at the electrode surface [45]. Copyright 2021, Nature. (C) Potential–current curves of photoanodes without the illumination (a), with illumination (b), and upon illumination without charge recombination (c). Copyright 2021, Nature [45]. (D) Schematic diagram of the moving direction of electrons and holes with and without an external magnetic field. Copyright 2021, Cell [99]. (E) Schematic of the bubbles dynamics, including generation, growth, and collapse [107]. Copyright 2021, Elsevier. (F) Selective heating on hot spots by a microwave field [99]. Copyright 2021, Cell.

Finally, raising the temperature can significantly inhibit the negative effects of byproducts on catalytic activities, which have been investigated in CO_2_ hydrogeneration. The continuous removal of byproducts adsorbed on the catalyst surface enables the refresh of active sites, thus effectively promoting the catalytic performance. Upon conducting CO_2_ hydrogenation, a small amount of water (5 or 10 µL) as a byproduct can effectively decrease the CH_4_ production rate within all temperatures (Figure 3A), indicating that the strong absorption of water molecules on a catalyst surface leads to the failure of the catalysts. However, an evident increase in CH_4_ production rate can be observed when the temperature increases to over the boiling point of water, which can be attributed to the desorption of water molecules from the catalyst surface [90]. For selective catalytic oxidation of NH_3_ (NH_3_‐SCO), a cost‐effective way to control NH_3_ pollution, the photothermal effect has been confirmed to significantly enhance the selectivity of targeted products. The cryptomelane nanowires were prepared for photothermal NH_3_‐SCO, and achieved an excellent NH_3_ (91.7%) conversion and N_2_ selectivity (94.7%). The study on the mechanism revealed that the photothermal effect enables the activation of NH_3_ into •NH_2_ by photogenerated holes, which was a key step in the photo‐iSCR mechanism (photo‐assisted internal selective catalytic reduction) [91].

### 3.2 Electrical Field Effects on Interfacial Charge Separation

According to the conformation of photocatalysts in electrolytes, photocatalysis can be divided into two types: (1) powder suspension system, and (2) photoelectrode system. Since the photoelectrode system needs an input of external electrical energy, it is denoted as a PEC system [92]. The advantage of the PEC system compared with the powder suspension system is the outstanding charge separation efficiency due to the tunable intensity of the built‐in electric field [93]. Considering an example of photoanodes (n‐type semiconductors) utilized in water oxidation semi‐reactions, electrons flow away from the semiconductor/electrolyte when photoanodes are immersed in solution. Owing to a higher Fermi level of n‐type semiconductors in comparison to the redox potential and a new charge equilibration due to the electron migration, a SCL, a region that is depleted of electrons, is formed on the photoanode surface. At the same time, equal negative charges accumulate on the electrolyte near the electrode surface, thus forming the Helmholtz layer. The SCL gives a desired direction of movement for electrons and holes at the semiconductor/electrolyte interface, where electrons are transferred to the counter electrode, occurring in a reduction reaction, while holes migrate to the electrolyte, participating in the water oxidation reaction, as shown in Figure 3B [45]. In the dark, photoanodes require an external bias more than 1.23 V versus the RHE. In practice, an additional overpotential (*η*_s_) is required to overcome dynamic barriers (gray curve in Figure 3C). Under the irradiation of visible light, an applied external bias is decreased due to the generation of photogenerated electrons and holes, which provides an additional photovoltage to overcome the energy barrier. The blue curves in Figure 3C represent the ideal photocurrent density curve without the charge recombination process. However, owing to the intrinsic crystal defects and slow mobility of semiconductor materials, a variety of photocatalysts suffer from serious charge recombination, thereby necessitating greater overpotentials, as illustrated in an orange curve (Figure 3C) [45]. Therefore, suppressing the recombination of charge carriers is a promising strategy for improving photocatalytic activities.

The width of SCL is related to the degree of band bending, which indicates the ability of charge separation. The wider the SCL, the stronger the charge separation ability. On the positive influence of band bending on charge separation, an effective method to enhance charge separation is to broaden the width of the SCL. In the past decades, introducing external electronic energy (external applied bias) has been considered a significant method to regulate the width of SCL. By controlling the magnitude of applied voltage, we can achieve different charge separation capabilities and photocurrent densities. A detailed demonstration is discussed in Section 4.2. In conclusion, external electrical energy plays a vital role in suppressing charge separation in a PEC device. The intensity of the built‐in electric field caused by the band bending is dependent on external electrical energy. In addition, the obtained current‐voltage curves provide a useful metric for initial assessment of photoelectrodes [94].

### 3.3 Magnetic Field Effects on Charge Separation

An external magnetic field usually has some significant effects on improving the charge separation efficiency and enhancing interfacial charge transfer. For some metal oxides and semiconductors, their resistance can vary with a changed applied magnetic field, which is denoted as the MR effect [95, 96]. MR effect is classified into two types: (1) positive MR; (2) negative MR. The materials with positive MR indicate that their MR enhances as the external magnetic intensity increases. On the contrary, negative MR implies that the MR decreases as the external magnetic intensity increases [97]. It is obvious that the semiconductor with negative MR can effectively promote charge carrier transport due to the reduced interfacial resistance. Three basic theories, including the Kondo effect [98], the spin‐glass state [99], and the Weyl effect [100], can explain the materials exhibiting a strong negative MR effect. Among these theories, the Kondo effect is a well‐developed theory through systematic and comprehensive research. According to the existing research results, the scattering of trace magnetic impurities in nonmagnetic metals may contribute to the Kondo effect [101]. At a low temperature, the interaction between electrons in the CB of nonmagnetic metals and localized magnetic moments of magnetic impurities reduces the resistance [102]. In addition to the MR effect caused by an applied magnetic field, the introduction of an external magnetic field also influences the practical movement of photogenerated electrons and holes by the Lorentz force. As illustrated in Figure 3D, moving electrons and holes in an applied magnetic field migrate to opposite directions, significantly inhibiting the recombination of the electron–hole pairs. Therefore, the Lorentz force arising from a magnetic field is expected to regulate the moving direction of charge carriers, further boosting the charge separation efficiency and photocatalytic activities.

Another important effect of a magnetic field on the photocatalytic performance is spin polarization. Since the photocatalytic activity is heavily dependent on its electronic structure, the change of spin state can manipulate the charge recombination process. For example, the photoexcited electrons with a spin‐up state are transferred to CB, while leaving the holes with a spin‐up state in VB. In this case, the electrons and holes tend to recombine due to the singlet state, that is, the total spin number is equal to 0. When a small amount of magnetic energy is injected into photocatalysts, the spin direction of electrons changes to the spin‐up state, which is identical to the spin direction of holes. Owing to the trilinear state (the total spin number is equal to 0), the recombination of the photogenerated electrons and holes is restricted [103]. Additionally, spin polarization can promote the orbital interaction between the active sites on the catalyst surface and reactants/intermediates. The strong interaction induces the formation of the reactive oxygen species on the catalyst surface, thus effectively enhancing the reactive activity [104].

### 3.4 Mechanical Field Effects on Reaction Kinetics and Charge Separation

Mechanical energy is commonly introduced into a photocatalysis system through high‐frequency ultrasonic vibration. Ultrasonic radiation can produce a “cavitation effect” in the liquid, which has a significant effect on mass transfer and chemical reactions [105]. Cavitation effect refers to the formation, growth, and collapse of gas or bubbles within the liquid. Under ultrasonic radiation, the liquid is subjected to a rapid change in pressure, resulting in localized low‐pressure zones where the liquid pressure is lower than its vapor pressure. Therefore, a variety of microbubbles are produced in the liquid [106]. The schematic diagram of the bubble dynamics is shown in Figure 3E, in which the microbubbles will undergo formation, expansion, oscillation, and collapse in the liquid phase. After the bubbles burst, they release a significant amount of energy, which can produce effects including intense local heating, shock waves, shearing force, and even high‐speed liquid jets. The local pressure and temperature can even reach 500 atm and 5000 K. The input of huge mechanical energy is beneficial for difficult surface chemical reactions with a high barrier. It can break the O–H bond in the H_2_O molecules and produce hydroxyl radicals, which are the crucial intermediate active substance for degrading organic pollutants. In addition, the shearing force of the ultrasonic wave can effectively inhibit the particle aggregation in liquid, thus creating more active sites [107].

The above‐mentioned mechanism is based on the influence of the cavitation effect on the liquid phase. For some piezoelectric materials with nanorod‐like structures or 2D‐dimensional nanosheet‐like structures, ultrasonic vibration leads to the mechanical deformation of materials by external stress. Then, the mechanical deformation can create a built‐in polarizing electronic field, thus promoting effective charge separation and transfer. The polarizing electronic field originates from deviations in the crystal centers of the positive and negative charges under ultrasonic radiation. The past applications for mechanical energy mainly focused on ultrasonic fields, but large‐scale commercialization is difficult due to the high intensity of energy input. Therefore, developing other forms of mechanical strain is the current research hotspot. Our group successfully introduced Bi vacancies into the bismuth vanadate (BiVO_4_) lattices and created a lattice strain by using a metal‐organic deposition method. Under AM 1.5 G illumination, the photocurrent density of a strain‐induced BiVO_4_ photoanode reached 6.20 mA cm^−2^ at 1.23 V vs. RHE. Density functional theory (DFT) revealed that lattice strain induced by the Bi vacancies causes the deformation of a small number of VO_4_ tetrahedra and creates a built‐in electric field, effectively promoting photogenerated electron–hole pair separation [108]. Compared with conventional mechanical energy induced by external ultrasonic waves, constructing a strain field by introducing atom defects may be a more practical and economical strategy.

### 3.5 Microwave Field Effects on Reaction Kinetics

MW radiation is a common rapid heating method, which has a higher thermal efficiency and shorter reaction time compared with conventional heating methods. Therefore, MW radiation is widely and frequently used in the field of fast synthesis, especially for high‐volume synthesis in a short time, such as covalent organic framework [109], and metal oxides [110]. Owing to the fast thermal effect, MW radiation can also be directly applied in photocatalytic reactions composed of organic pollutant degradation and organic synthesis. The improved photocatalytic performance originated from thermal and nonthermal effects caused by an external MW field. Previous studies have distinguished thermal and nonthermal effects through introducing a cooling system and confirmed that non‐thermal effects can effectively enhance the organic degradation efficiency [44]. However, it is difficult to investigate the influence of non‐thermal effects on semiconductor properties and chemical reaction kinetics.

In this article, we mainly discuss the beneficial effects of thermal effects on photocatalysis. Similar to thermal‐assisted photocatalysis, microwave‐assisted photocatalysis using thermal effects makes full use of the thermal energy to affect the photocatalysis process. The difference is that heat conduction in microwave‐assisted photocatalysis is faster than that in thermal‐assisted photocatalysis. According to the selective and fast heating of a MW field, the hotspot effect has been proposed to demonstrate the microwave‐assisted mechanism. As shown in Figure 3F, a variety of catalytically active sites are located on the photocatalyst surface. Traditional photothermal effect can homogeneously heat the whole photocatalyst particle; in contrast, the microwave‐assisted heating strategy only quickly heats the local surface hotspots instead of the whole particle. Therefore, some side reactions can be significantly inhibited [99]. As an example, via microwave‐assisted selective heating, polymeric nickel and iridium dual catalysts can selectively conduct Buchwald–Hartwig‐type amination of aryl chlorides and obtain a high conversion rate. Additionally, tremendous MW energy can break the hydrogen bonds between water molecules, enhancing the desorption of water molecules at the semiconductor surface. The boosted water molecules desorption is favorable for the exposure of active sites at the surface, improving chemical reaction kinetics [111].

In summary, external energy fields play an important role in enhancing charge separation, improving surface reaction kinetics, and increasing selectivity of the target products. For the thermal field, the elevated temperature on the surface of photocatalysts by the photothermal effect can significantly inhibit the recombination of the photogenerated carriers, improving the photocatalytic efficiency with an increased mobility of charge carriers. Additionally, by regulating the magnitude of the temperature, the selectivity for target products can be increased. However, elevated temperatures may destabilize photocatalysts, causing sintering, phase changes (e.g., anatase‐to‐rutile in TiO_2_), or reduced active sites. For the electric field, externally applied voltages change the charge distribution of the SCL. The built‐in electric field induced by a suitable bias enables a strong separation of photogenerated carriers on SCL, where holes are transferred to the active sites, and electrons are transferred to the counter electrode. MR effect, Lorentz force, and spin polarization caused by a magnetic field have been considered as effective methods to control the motion behavior of charge carriers. Especially for the Lorentz force, it makes electrons and holes migrate in opposite directions, resulting in a rapid separation of photogenerated carriers. The current challenge in magnetic‐field‐assisted photocatalysis is the rational reactor design. Developing a uniform magnetic field distribution across the reactor is still difficult. Ultrasonic vibration generally leads to a cavitation effect in the liquid phase and mechanical deformation of materials, introducing a mechanical field. The cavitation effect releases tremendous energy by the collapse of microbubbles, which could activate the intermediate active substance. Mechanical deformation of a special nanostructure generates a built‐in polarizing electric field, leading to a favorable separation of charge carriers. Similar to a magnetic field, the reasonable design of an external device that can produce uniform ultrasonic vibration without damage to the materials themselves is a challenge for mechanical‐field‐assisted photocatalysis. For an MW field, it can heat local materials rapidly and selectively instead of the whole materials, called the hotspot effect. The microwave‐assisted selective heating strategy is beneficial for activating the intermediate products and reducing the reaction barrier. Nevertheless, uniform MW distribution is difficult to achieve in large‐scale reactors, which limits its commercial applications in the future. For commercial applications, magnetic‐field‐assisted photocatalysis has the potential due to its scalability and recyclability. For high‐efficiency, cutting‐edge applications, MW and electric fields show greater potential if material and reactor challenges are addressed. Ultimately, hybrid systems integrating multiple fields will likely dominate the future of photocatalysis, balancing efficiency, cost, and sustainability.

## 4 Emerging External‐Energy Integrated Photocatalysis

### 4.1 Thermal Energy

Thermal energy has been the most frequently used in diverse fields, such as energy conversion and chemical reactions. Recently, thermal energy has been introduced into the field of photocatalysis and exhibits a remarkably improved photocatalysis performance. Enhanced performance caused by thermal energy can be attributed to two important mechanisms. On the one hand, the thermal energy enhances the kinetic driving force and facilitates mass transformation by improving the charge carrier mobility and the population of electrons in the excited state, thus speeding up the reaction process [112]. For instance, a hollow core‐shell Au/g‐C_3_N_4_@Ag_3_PO_4_ photocatalyst exhibited a higher hydrogen evolution rate and tetracycline degradation efficiency in comparison to the pristine g‐C_3_N_4_ counterpart, which can be attributed to a local high‐temperature environment through the hollow core‐shell structure where the carrier separation ability of the Au/g‐C_3_N_4_@Ag_3_PO_4_ photocatalyst can be effectively enhanced [113]. On the other hand, the increase in the reaction temperature can tune the redox potential of semi‐reactions, which can broaden the application range of narrow‐band‐gap semiconductors. As an example, Han and coworkers predicted temperature‐dependent behavior of redox potentials on water splitting, ammonia synthesis, and carbon dioxide reduction reactions over a temperature range from 298.15 to 1500 K by using a simple thermodynamic approach. It was found that NH_3_ synthesis and CO_2_ reduction exhibited varying redox potentials with a gradually increased reaction temperature, and the corresponding experiments also confirmed this result [114]. In a thermal‐assisted photocatalytic system, thermal energy can be obtained from either an external heating field or a photothermal effect of the photothermal catalyst, which can convert the solar light into thermal energy [115]. The light‐to‐heat conversion abilities of various materials are primarily determined by the responses of their electronic or bandgap structures to light radiation. There are three fundamental mechanisms: (1) plasmonic photothermal effect; (2) nonradiative relaxation in the excited semiconductors; and (3) thermal vibrations of molecules in a light‐to‐heat conversion process [116].

**FIGURE 4:**
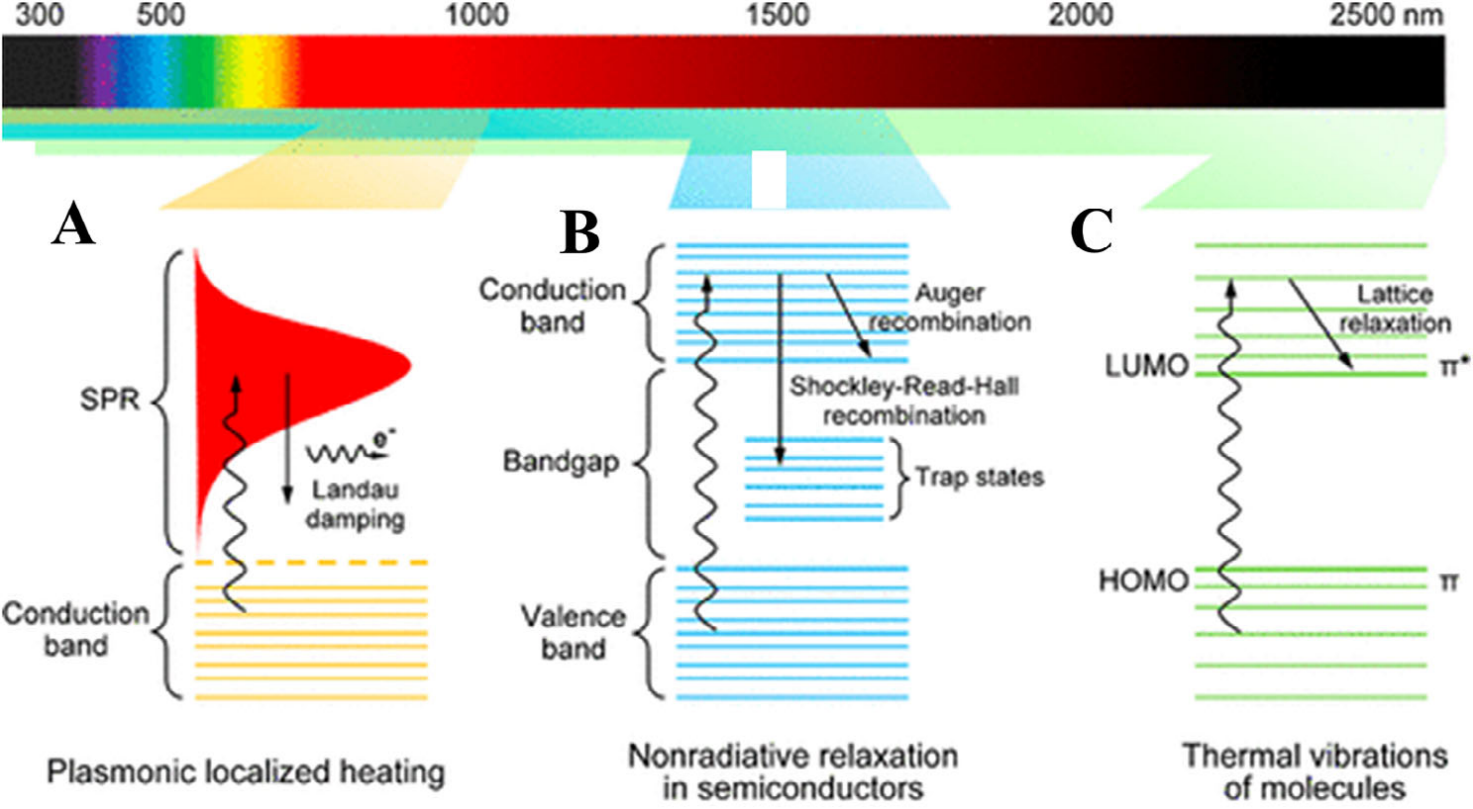
Three mechanisms of the photothermal effect with a corresponding light absorption range. (A) Plasmonic localized heating. (B) Nonradiative relaxation in semiconductors. (C) Thermal vibrations of molecules [118]. Copyright 2022, Wiley.

The LSPR effect, an optical phenomenon appearing in metallic structures down to the subwavelength‐sized dimensions, refers to the collective oscillations of the conduction‐band electrons restricted inside highly conductive nanostructures. These oscillations originate from the interaction of light with the conducting NPs smaller than the incident wavelength [117]. When the frequency of the input photon coincides with the overall vibrational frequency of the electrons at the surface of the NPs, LSPR excitation in metallic nanostructures can contribute to the absorption of photons, especially in the NIR region (Figure 4A) [118]. After an LSPR excitation process, relaxation typically takes place either through radiative photon re‐emission (scattering) or non‐radiative excitation of energetic hot charge carriers. The hot charge carriers then collide with low‐energy electrons and convert the electron energy into heat energy. At the same time, low‐energy electrons couple with the metallic lattice through electron–phonon scattering, leading to a lattice thermalization of the nanostructure [119].

For non‐plasmon semiconductors, the photothermal effect can also arise in the form of nonradiative relaxation. Under light irradiation, the non‐plasmon semiconductor absorbs photons larger than its bandgap energy, which excites electrons to jump from the VB to the CB, even to energy levels above the CB minimum. Afterward, the electrons return to the edge of the CB by nonradiative relaxation and release thermal energy, leading to a localized temperature increase in the lattice (Figure 4B) [120]. Unlike LSPR and nonradiative relaxation, thermal vibrations of molecules tend to occur in carbonaceous and polymeric materials with abundant conjugated π‐bonds. Therefore, conjugated π‐bonds play a vital role in the thermal vibration mechanism of molecules. Upon photon absorption, the loosely held electrons in the conjugated system can be excited from the ground state (the highest occupied molecular orbital (HOMO)) to a higher energy state (the lowest unoccupied molecular orbital (LUMO)) [121]. Then the excited electrons are restored to the ground state through vibrational‐electronic coupling, which releases the excess energy in the form of thermal energy (Figure 4C). In addition, semiconductor materials with conjugated π‐bonds can exhibit increased surface interactions with the surrounding medium (such as water or biological tissues), which efficiently enhances heat transfer and the efficiency of photothermal conversion. The surface area and morphology of the conjugated systems, like in NPs, can be engineered to optimize these interactions [89].

Subsequently, we will introduce photothermal‐assisted photocatalysis based on the above‐mentioned three fundamental mechanisms. The involved applications of photothermal‐assisted photocatalysis include hydrogen production, CO_2_ reduction, and pollutant degradation. A variety of photothermal materials, such as noble NPs (Au NPs, Ag NPs), transition metal materials (MnO_2_, Co_3_O_4_), and carbon‐based materials (g‐C_3_N_4_, RGO), are also involved [122]. Their energy alignment, electronic structure, and optical properties can effectively convert light into thermal energy, thereby significantly improving charge transfer and reaction kinetics.

#### 4.1.1 Plasmonic Metals for Thermal‐Assisted Photocatalysis

Plasmonic metals with excellent light‐to‐heat conversion properties due to the LSPR effect have attracted a lot of attention in the field of photocatalysis. On the one hand, thermal energy is generated through the above‐mentioned mechanisms to increase the temperature of the system, thus accelerating the photocatalytic kinetics. On the other hand, the energetic hot carriers generated following the excitation can be adsorbed on reaction substrates to drive chemical reactions, thus significantly enhancing the reaction dynamics and reaction selectivity [120]. The LSPR effect has been found in a variety of semiconductor nanocrystals (NCs), including metal oxides (like WO_3‐x_, ZnO, In_2_O_3_) [123] and sulfides (like Fe_1–_ *_x_* S_2_, MoS_2_) [124]. LSPR can be turned across a wide range of the optical spectrum from visible to far‐IR through controlling the carrier concentration in the semiconductor NCs. For instance, Cu_7_S_4_ NCs showed strong NIR absorption and achieved a maximum photothermal conversion efficiency of 77.1% from self‐doping, which resulted from the LSPR effect of the Cu_2‐x_S NCs [125].

Since the LSPR effect only occurs when the surface plasmons are restricted within a small volume, only NPs with a small size that is comparable in size to the wavelength of the incident light can take advantage of the interaction between the solar light and the reaction substrates to improve the photocatalytic performance. The most widely and frequently used NPs are Au NPs and Ag NPs, which can absorb and scatter light throughout the visible and NIR regions (i.e., Ag NPs with the LSPR can absorb the light with a wavelength ranging from 300 to 1200 nm) [126]. Although Ag NPs exhibit an outstanding light absorption ability, stability is still a challenging issue because Ag NPs are susceptible to oxidation, leading to degradation of the plasmonic properties. An alternative solution is to use gold (Au) NPs, which exhibit excellent stability due to their chemically inert and oxidation‐free features. For example, Au NPs supported on two‐dimensional TiO_2_ nanoflakes show impressive photocatalytic performance for bio‐derived glycerol photothermal reforming hydrogen production [127]. The 2D Au/TiO_2_ nanoflake photothermal catalyst was fabricated by a hydrothermal and deposition precipitation method. A series of optical characterizations and kinetic calculations found that the introduction of Au NPs has a negligible effect on the bandgap of TiO_2_, and has a remarkable improvement for the transfer of photogenerated carriers. The loading of Au NPs constructs a Schottky barrier between the semiconductor surface and solution, which effectively inhibits the recombination of the photogenerated electron–hole pairs under ultraviolet light.

**FIGURE 5:**
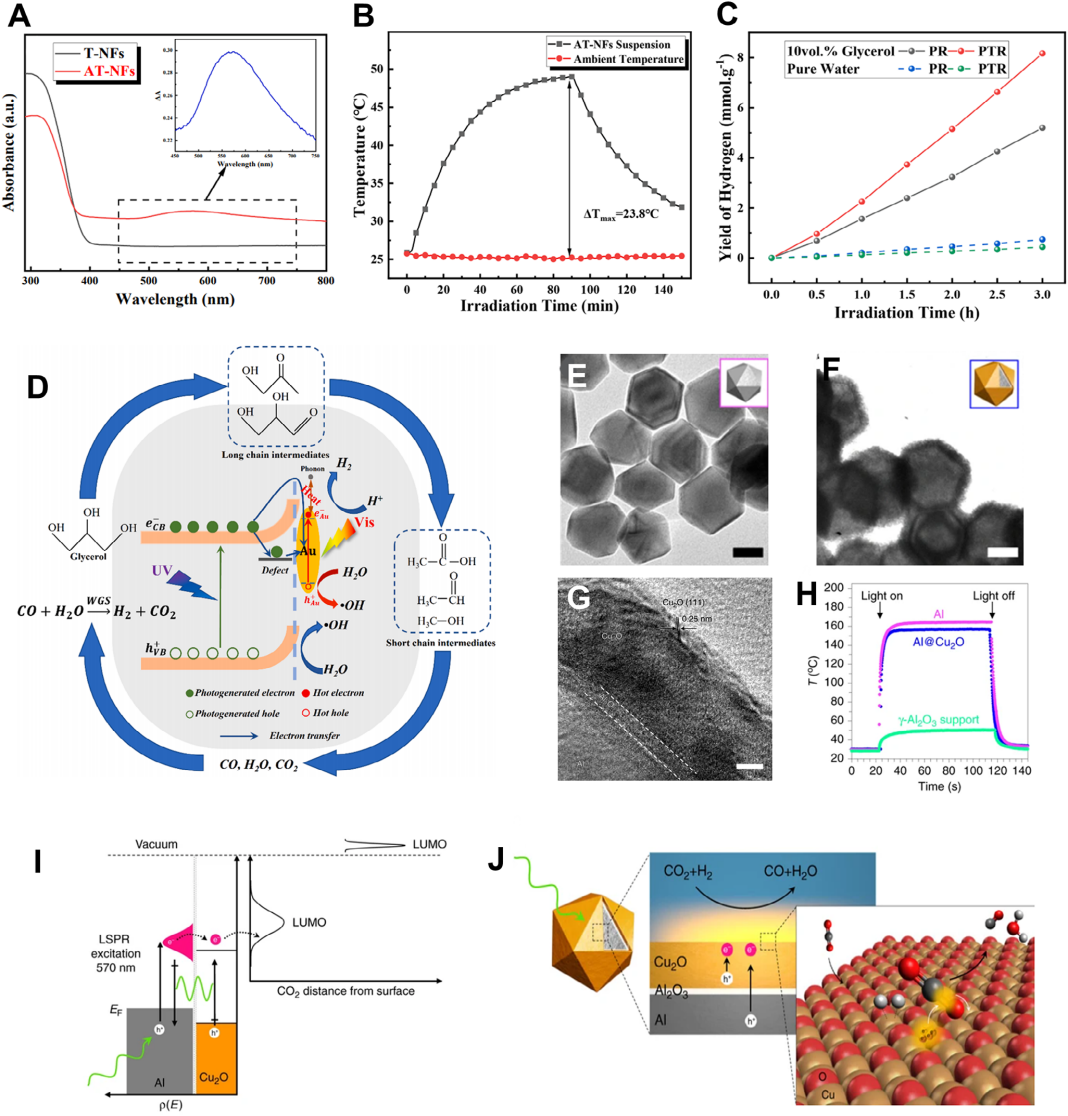
(A) UV–Vis plot of TiO_2_ nanoflakes (T‐NFs) and Au decorated TiO_2_ nanoflakes (AT‐NFs). (B) Temperature curves of AT‐NFs and ambient temperature within 150 min. (C) Photocatalytic performance of glycerol reforming in 10 vol% glycerol and pure water during 3 h irradiation. (D) Schematic mechanism of glycerol photothermal reforming and carrier migration route during irradiation [127]. Copyright 2022, Elsevier. TEM images of (E) pristine Al nanocrystals (Al NCs) and (F) Cu_2_O wrapped Al NCs. Scale bars in (E) and (F) are 50 nm. (G) HRTEM image of Al/Al_2_O_3_/Cu_2_O nanostructure. Scale bar in (G) is 5 nm. (H) Comparison of steady‐state temperature curves for oxide‐supported plasmonic nanoparticles and pure oxide with and without irradiation. (I) Schematic band structure of Al@Cu_2_O for plasmon‐mediated carrier generation for injection into the unoccupied state of CO_2_ for C–O bond activation. (J) Schematic illustration of plasmon‐induced carrier‐driven reverse water‐gas shift (rWGS) on Al@Cu_2_O [129]. Copyright 2017, Nature Publishing Group.

As shown in Figure 5A, the spectral absorption wavelength of TiO_2_ is less than 400 nm, while 2D Au/TiO_2_ nanoflake exhibits an additional peak between 500 and 650 nm, which can be attributed to the LSPR effect caused by Au NPs. This extended light absorption contributes to the increase in temperature. Under the radiation of an Xe lamp without a filter, the temperature of 2D Au/TiO_2_ nanoflake increases from 23.8°C to 48.1°C within 80 min, resulting in enhanced photocatalytic kinetics and reduced interfacial resistance (Figure 5B). The photocatalytic reforming performance of the 2D Au/TiO_2_ nanoflakes with and without photothermal effect is shown in Figure 5C. It can be observed that the hydrogen yield with the photothermal effect (8.2 mmol. G^−1^) is obviously higher than its counterpart without the photothermal effect (5.2 mmol. G^−1^) after reaction for 3 h. Under the whole process of glycerol photothermal reforming hydrogen production, as shown in Figure 5D, Au NPs play a crucial role in converting H^+^ to hydrogen. The formed Schottky barrier is favorable for the transfer of photogenerated electrons from the VB to Au NPs, in which H^+^ ions are reduced to hydrogen. In addition, hot carriers caused by the LSPR effect efficiently facilitate each reaction pathway, including vital conversion from long‐chain intermediates (such as glyceraldehyde) to short‐chain intermediates (such as methanol, acetaldehyde, and carboxylic acid) [127].

Currently, earth‐abundant metals, including Al and Cu, have shown the potential to replace Au and Ag as photothermal catalysts because of their LSPR performances comparable to those of Au and Ag while having a lower cost. Al nanostructures exhibit an excellent light‐to‐heat conversion efficiency in the UV–Vis region due to the strong plasmonic effect [128]. Although Al is highly susceptible to being oxidized, the passivation layer of aluminum oxide formed can effectively inhibit the failure of the LSPR effect. An embedded aluminum in cuprous oxide antenna‐reactor heterostructures (Al@Cu_2_O) for the reverse water‐gas shift (rWGS) reaction under milder illumination was reported. The geometry of the Al@Cu_2_O antenna reactor takes advantage of the LSPR effect of aluminum to provide energetic hot carriers. Figure 5E–G clearly shows the TEM images of the Al NPs with and without a Cu_2_O shell around the Al core. Under the illumination of visible light with a light intensity of 10 W cm^−2^, the surface temperature on the γ‐Al_2_O_3_ catalyst is lower than 60°C (Figure 5H). After decorating some plasmonic NPss into the pure oxide support, the average surface temperature increases to 150°C, which is 2.5 times higher than that of the pure γ‐Al_2_O_3_. Consequently, the outstanding photothermal performance can be attributed to the photothermal energy from electron–electron scattering in the hot‐carrier decay pathway. Compared to conventional photothermal materials, such as Ag NPs and Au NPs, Al has a higher Fermi level and a suitable band alignment so that plasmon‐induced hot electrons can transfer from Al to the Cu_2_O layer (Figure 5I) with a non‐competing direct excitation pathway (interband transitions) at higher photon energies. It is vital for driving difficult chemical reactions (i.e., carbon dioxide reduction and artificial nitrogen fixation reactions), in which antibonding orbitals of these molecules are only accessible by highly energetic carriers [129].

The most difficult step in plasmon‐induced carrier‐assisted rWGS is CO_2_ activation, which can be attributed to the difficult electron injection into CO_2_ caused by the negative electron affinity (−0.6 eV±0.2 per CO_2_ molecule in gas‐phase). The introduction of Cu_2_O is in favor of the charge redistribution, thereby enhancing carrier transfer from the metal surface to the absorbed CO_2_, and reducing the energy barrier for transient electron transfer to unoccupied states of the adsorbed CO_2_ (Figure 5J) [129]. Recently, plasmon hot carriers induced by LSPR effects have been confirmed to be favorable for photocatalytic reactions, especially for CO_2_ reduction. A plasmonic nanohybrid system comprising NiO/Au/Re^I^(*phen‐NH_2_*)(CO)_3_Cl (*phen‐NH_2_*  =  1,10‐phenanthrolin‐5‐amine) was designed to reduce CO_2_. The molecular catalyst (Re^I^(*phen‐NH_2_*)(CO)_3_Cl) on the plasmonic Au surface effectively facilitates the selective extraction of hole electrons and catalytic performance. Mechanism research reveals a sequential reduction of the rhenium complex with two protonations between these reduction steps [130].

#### 4.1.2 Transition Metals for Thermal‐Assisted Photocatalysis

Most of the photocatalytic semiconductors can only respond to UV light due to the wide bandgap, while the visible‐NIR region, which accounts for the majority of the solar spectrum, is not fully utilized. Loading transition metal materials with a strong photothermal effect on semiconductors can effectively expand the utilization of the solar spectrum from UV light to NIR light. Owing to the extended light absorption, photoinduced non‐radiative relaxation of the charge carriers upon irradiation of solar light can convert the light energy into thermal energy, thus increasing the surface temperature of the photocatalysts. In addition, suitable band alignments between transition metals and semiconductors can facilitate charge separation and transportation. For example, graphitic carbon nitride (g‐C_3_N_4_), a promising non‐metallic organic photocatalyst, has attracted extensive attention due to its unique electronic structure and light absorption capacity in the visible region [99, 131]. However, rapid charge recombination and inadequate visible light absorption limit its applications.

**FIGURE 6:**
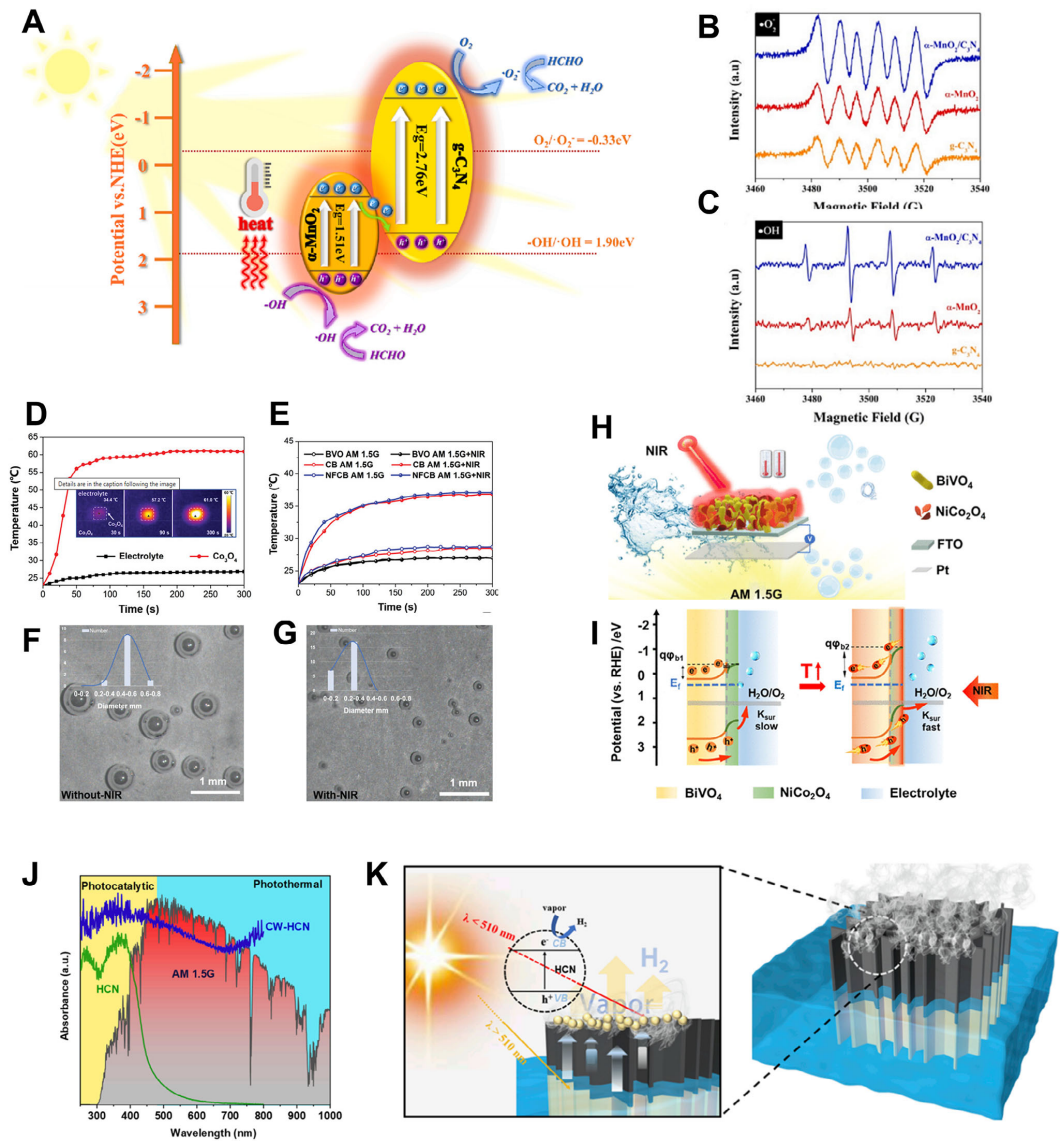
(A) Band alignment and reaction mechanisms of α‐MnO_2_/g‐C_3_N_4_ for the degradation of HCHO under solar‐light irradiation; DMPO spin‐trapping EPR signals of (B) ·O_2_^–^ and (C) ·OH over g‐C_3_N_4_, α‐MnO_2,_ and α‐MnO_2_/g‐C_3_N_4_ composite [136]. Copyright 2023, Elsevier. (D) Time‐dependent temperature curves of pure Co_3_O_4_ and electrolyte. (E) Time‐dependent temperature curves of BiVO_4_, Co_3_O_4_/BiVO_4_, and NiOOH/FeOOH/Co_3_O_4_/BiVO_4_ photoanodes under AM 1.5 G illumination with or without constant NIR light irradiation. (F) Digital images of the NiOOH/FeOOH/Co_3_O_4_/BiVO_4_ composite photoanode and corresponding size distribution of generated oxygen bubbles: (F) without NIR irradiation, and (G) with NIR irradiation [141]. Copyright 2021, Wiley. (H and I) Mechanism of the improved PEC performance of the NiCo_2_O_4_/BVO photoelectrode through the photothermal effect [142]. Copyright 2021, American Chemical Society. (J) Ultraviolet–visible diffuse reflection spectra (UV–Vis DRS) of carbonized wood‐protonated g‐C_3_N_4_ (CW‐HCN) and pure HCN; (K) Schematic diagram of the combined photothermal–photocatalytic water steam evaporator to produce hydrogen [145]. Copyright 2023, Elsevier.

MnO_2_, a narrow band gap transition metal oxide, has been proven to be an excellent photothermal material. Rong and coworkers grew α‐MnO_2_ nanowires on the surface of 2D g‐C_3_N_4_ nanosheets, forming a Z‐scheme heterojunction. The as‐synthesized α‐MnO_2_/g‐C_3_N_4_ composites have a higher photocatalytic efficiency for degrading formaldehyde (HCHO) at room temperature compared to their counterpart (pure g‐C_3_N_4_). Schematic mechanism of the HCHO degradation (Figure 6A) clearly reveals that the photogenerated holes in g‐C_3_N_4_ were not able to generate ·OH, and the photogenerated electrons in α‐MnO_2_ cannot effectively produce ·O_2_^−^ radicals due to the thermodynamic restriction. When combining α‐MnO_2_ with g‐C_3_N_4_ to construct a Z‐scheme heterojunction [132, 133, 134, 135], the enhanced thermal energy caused by the photothermal effect enables an obvious ·OH signal on g‐C_3_N_4_, and ·O_2_^−^ signal on α‐MnO_2_ in the electron paramagnetic resonance (EPR) spectra (Figure 6B,C), achieving a complete conversion of HCHO under solar illumination with an intensity of 191 mW/cm^2^. Consequently, the thermal energy generated from light radiation can accelerate the migration of the photogenerated electrons and restrict the recombination of the photogenerated electron–hole pairs. On the other hand, the photothermal effect can decrease the activation energy of lattice oxygen, resulting in the generation of more surface‐active oxygen species [136].

Recently, the minority carrier hopping mechanism occurring in some semiconductors (i.e., BiVO_4_ [137], TiO_2_ [138], and α‐Fe_2_O_3_ [139]) has been proven to be activated at an elevated temperature. The introduction of transition metal oxides as a photothermal conversion material is a promising strategy to make full use of the activated minority carrier hopping mechanism, thereby effectively improving interfacial charge transfer and chemical reaction kinetics simultaneously. The judiciously designed NiOOH/FeOOH/Co_3_O_4_/BiVO_4_ photoanode, in which the Co_3_O_4_ is a photothermal layer inserted between BiVO_4_ and the surface co‐catalyst (NiOOH/FeOOH) [140], exhibits an outstanding photocatalytic performance with a photocurrent density of 6.34 mA cm^−2^ at 1.23 V_RHE_. Time‐dependent temperature curves of Co_3_O_4_ and the electrolyte solution in Figure 6D indicate that Co_3_O_4_ has an excellent photothermal effect, in which the temperature of the Co_3_O_4_ layer increases from 23 to 60°C within 60 s. After loading the Co_3_O_4_ layer on BiVO_4_ photoanodes, the Co_3_O_4_/BiVO_4_ also exhibits a remarkable photothermal effect in comparison to pure BiVO_4_ photoanodes (Figure 6E). Another noteworthy point is the influence of the photothermal effect on the release of formed oxygen bubbles, which is an important process in the water oxidation reaction. In the case of NiOOH/FeOOH/Co_3_O_4_/BiVO_4_, the photoanodes with photothermal effects have a smaller bubble adhesion and radius under the irradiation of an NIR light, which is favorable for the removal of bubbles (Figure 6F,G) [141]. Except for Co_3_O_4_, NiCo_2_O_4,_ as a spinel‐type metal oxide, can also significantly improve water oxidation activity when photoanodes are irradiated by NIR light (Figure 6H). The mechanism for the significantly improved PEC performance due to the photothermal effect is illustrated in Figure 6I. Under the elevated temperature, the photogenerated electrons and holes can be quickly transferred from the BiVO_4_ bulk to NiCo_2_O_4_, effectively restricting the recombination of the photogenerated carriers [142].

#### 4.1.3 Carbon‐Based Materials for Thermal‐Assisted Photocatalysis

Carbon‐based materials, including graphite, carbon nanotubes, and graphene, are common photothermal materials with a wider spectral absorption range and higher photothermal conversion efficiency compared to transition metal materials. Conjugated π‐bonds in carbon‐based materials can significantly enable efficient charge transport due to the improved mobility of delocalized π‐electrons under an evaluated temperature [143]. A widely and frequently used carbon‐based material is g‐C_3_N_4_, which can significantly convert photon energy to thermal energy. Since the distinctive thermal vibration mechanism of the carbon‐based material, photogenerated electrons that are excited to a high level will return to the ground state by a non‐radiative relaxation and release thermal energy [144]. Furthermore, the localized temperature increases due to its low thermal conductivity, leading to improved chemical reaction kinetics. For instance, spherical g‐C_3_N_4_ (HCN) was successfully grown on a carbonized wood (CW) by a one‐step hydrothermal method. The CW‐HCN system was able to facilitate the conversion of water to steam production due to the extended light absorption capability. CW, as an excellent solar steam evaporator, can broaden the absorption wavelength of the solar spectrum. As shown in Figure 6J, ultraviolet–visible diffuse reflection spectra (UV–Vis DRS) clearly exhibit that the CW‐HCN evaporator has an absorption wavelength of the solar spectrum from visible to near‐IR regions. When the CW is irradiated by the solar light, transformed thermal energy by the photothermal effect can generate water steam, which is transferred to the surface g‐C_3_N_4_ photocatalysts to participate in hydrogen production (Figure 6K). This combination of photothermal evaporator and photothermal‐assisted photocatalysts efficiently utilizes IR light to drive water oxidation and achieve hydrogen production from seawater [145].

Another carbon‐based material used in photothermal‐assisted photocatalysis is reduced graphene oxide (RGO), a semiconductor material that can absorb the full‐spectrum solar light. Owing to an outstanding light absorption ability and catalytic activity, it can be applied to participate in CO_2_ reduction to produce high‐value fuels, such as hydrocarbons, aldehydes, and alcohols. Shang and coworkers designed an RGO/protonated g‐C_3_N_4_ (H‐CN) photocatalyst with a two‐dimensional S‐scheme heterostructure. Upon a 5‐h visible irradiation, RGO/H‐CN exhibits outstanding thermal‐assisted photocatalytic reduction performance with the product yields of CO and CH_4_ being 10.21 and 5.56 µmol/g, respectively. In conclusion, the improved CO_2_ reduction performance can be ascribed to the photothermal effect of RGO, which not only promotes the separation of photogenerated electron–hole pairs but also accelerates molecular motion and enhances the adsorption/activation of CO_2_ [146]. The stability of photocatalysts during high‐temperature photothermal conditions remains challenging. CuFe_2_O_4_@C/Cd_0.9_Zn_0.1_S (CFO@C/CZS) S‐scheme photocatalysts showed an excellent photothermal effect upon irradiation of visible light, by which the temperature of the optimized CFO@C/CZS photocatalyst increased from room temperature to a high temperature (41.6 C). After 16 h cycle experiment, its hydrogen evolution efficiency remained stable. Morphological and structural characterization illustrated that the optimized CFO@C/CZS photocatalysts exhibited an unchanged crystal structure and elemental composition under a high‐temperature condition induced by the photothermal effect [147].

### 4.2 Electrical Energy

**FIGURE 7:**
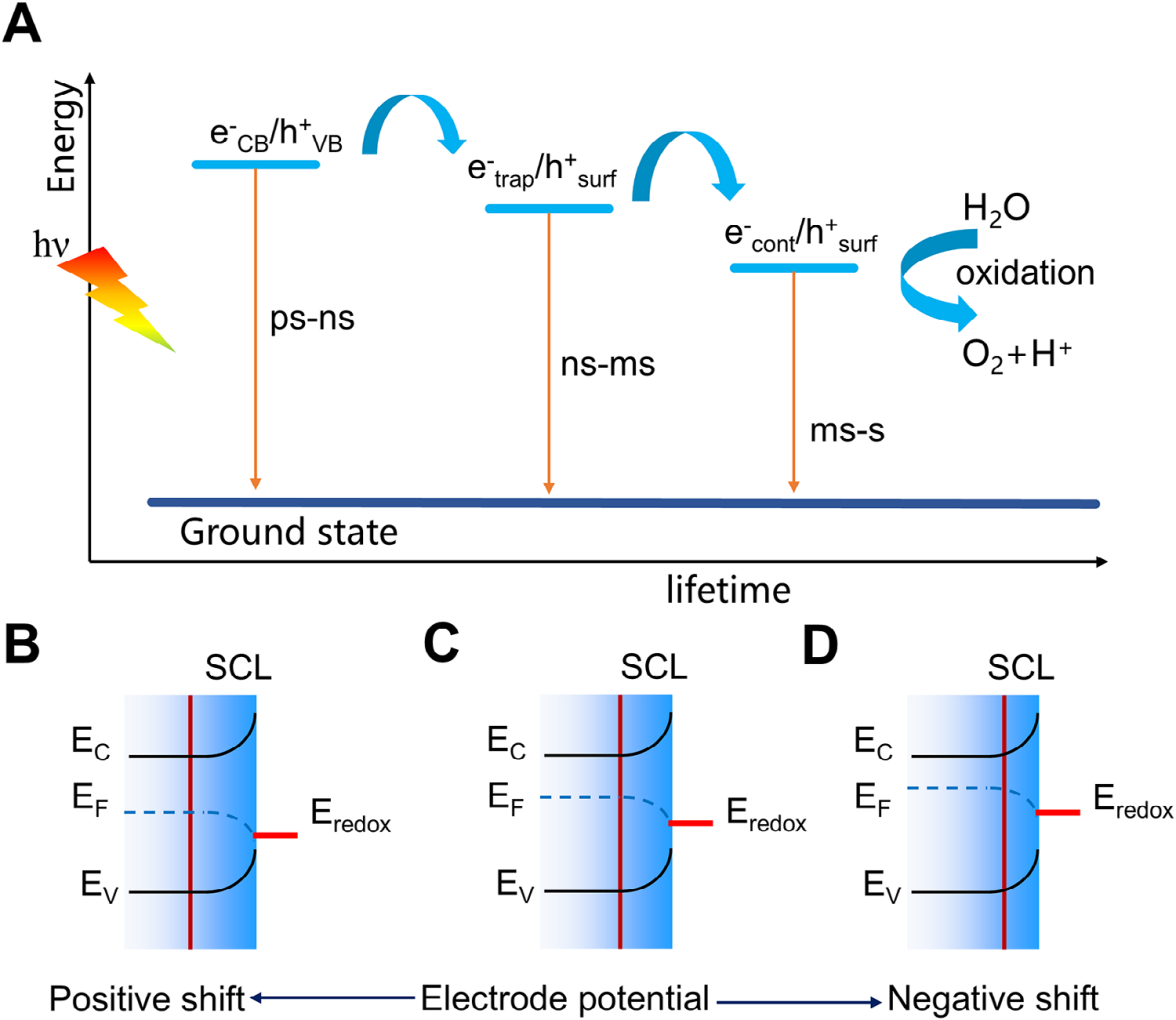
(A) Schematic illustration of energy loss associated with the carrier lifetime for a photocatalysis process. Schematic diagrams of the band position change with the variation of electrode potentials. (B) The electrode potential shifts positively. (C) initial electrode potential. (D) The electrode potential shifts negatively.

Driving redox reactions using photocatalytic semiconductor materials can be divided into a series of steps with various timescales. Electrons in the filled VB are excited to the empty CB and leave the same amount of holes in the VB with a timescale of ps‐ns. Then, photogenerated charges strongly interact with the lattice to form polaron states, which is sometimes denoted as a self‐trapping process. Finally, the trapped photogenerated charges are extracted to the semiconductor surface, where the water redox reactions occur. As shown in Figure 7A, the photoinduced electrons and holes in an excited state need at least half of the energy to achieve the whole charge separation process. At the same time, there are multiple charge recombination pathways resulting in the loss of charges under the excited state (decay to ground state) over broad timescales (from ps to s), including both bulk and surface recombination. The kinetic competition between charge generation and recombination has been considered as the biggest challenge for developing highly efficient semiconductor materials and the photocatalytic water splitting system. Owing to the built‐in electric field of semiconductor electrodes that can efficiently separate the photogenerated electron–hole pairs and inhibit surface recombination, the PEC system was introduced to photocatalytic water splitting and exhibited a high STH efficiency compared to conventional powder‐based photocatalytic systems [45].

In this section, we will illustrate the structures of a built‐in electric field in metal oxide photoelectrodes, demonstrating mechanisms of separating electron–hole pairs at the photoelectrode surface by the interfacial SCL. In addition, some regulatory methods that can strengthen the electric field, including increasing the external bias, loading a charge‐extracted layer, and designing surface co‐catalysts, are also involved.

#### 4.2.1 External Applied Bias

When semiconductor electrodes (solid phase) contact with electrolyte (liquid phase), the Fermi level of a semiconductor surface (*E*_F_) equals the water redox potential (*E*_redox_) if the semiconductor‐electrolyte interface achieves equilibration by the flow of electrons and holes. The loss of carriers at the semiconductor surface results in the formation of the SCL, a region that can generate the band bending [148]. At the electrolyte side, a double electric layer similar to a metal electrode is created due to the electrostatic effect, consisting of the Helmholtz layer and the Gouy–Chapman layer. The band bending at SCL creates a built‐in electric field that usually promotes the separation of photogenerated electron–hole pairs. An n‐type photoanode has a Fermi level (*E*_F_) that is close to the CB and higher than the E_redox_, which makes the electrons transfer from the semiconductor to the electrolyte and leave positive charges at the SCL region. Owing to the electrostatic effect, large amounts of anions with negative charges accumulate on the electrolyte side of the semiconductor/electrolyte interface. Therefore, a built‐in electric field across the SCL and semiconductor/electrolyte interface with a direction to the electrolyte is formed. Typically, the built‐in electric field has a potential difference over 10^5^ V cm^−1^ and carrier mobility between 10–1000 cm^2^ V^−1^ s^−1^, leading to an excellent charge separation ability compared to the particle‐based photocatalysis system [149].

The SCL thickness and the degree of band bending play a vital role in promoting the photogenerated charge separation at a photoelectrode surface. The thicker the SCL, the greater the electric field strength in the built‐in electric field created by the SCL. Regulating externally applied bias has been considered a simple and effective method to change the SCL thickness and the degree of band bending. Applying a positive potential on a photoelectrode corresponds to extracting electrons from a photoelectrode surface (anodic polarization). On the contrary, applying a negative potential on a photoelectrode is equal to injecting electrons into the photoelectrode surface (cathodic polarization). The extraction and injection of carriers causes the change of the Fermi level and band bending in the SCL region.

Figure 7B–D qualitatively exhibits the effects of externally applied potentials on the band bending and SCL thickness for an n‐type photoanode. The change in electrode potentials for an ideal non‐degenerate semiconductor electrode usually causes the variation of the Fermi level in the SCL, and the Fermi level in the semiconductor bulk remains unchanged because the SCL can bear almost all of the external potentials. Once the external potential is negatively shifted due to the injection of photogenerated electrons, the Fermi level in the SCL increases to a high level, leading to a decreased degree of band bending and a shortened SCL thickness (Figure 7D). It is unfavorable for the separation of electron–hole pairs at the photoelectrode surface because the shortened SCL thickness weakens the built‐in electric field intensity. Conversely, the positive shift of external applied bias by extracting electrons from the SCL generally increases the degree of band bending and the SCL thickness, which results in greater built‐in electric field strength (Figure 7B), where the photogenerated electron–hole pairs can be easily separated at the photoelectrode surface. Additionally, an enhanced electric field provides enough driving force for extracting holes from the electrode surface to the double electric layer in which the water oxidation reaction occurs.

#### 4.2.2 Charge‐Extraction Layers

**FIGURE 8:**
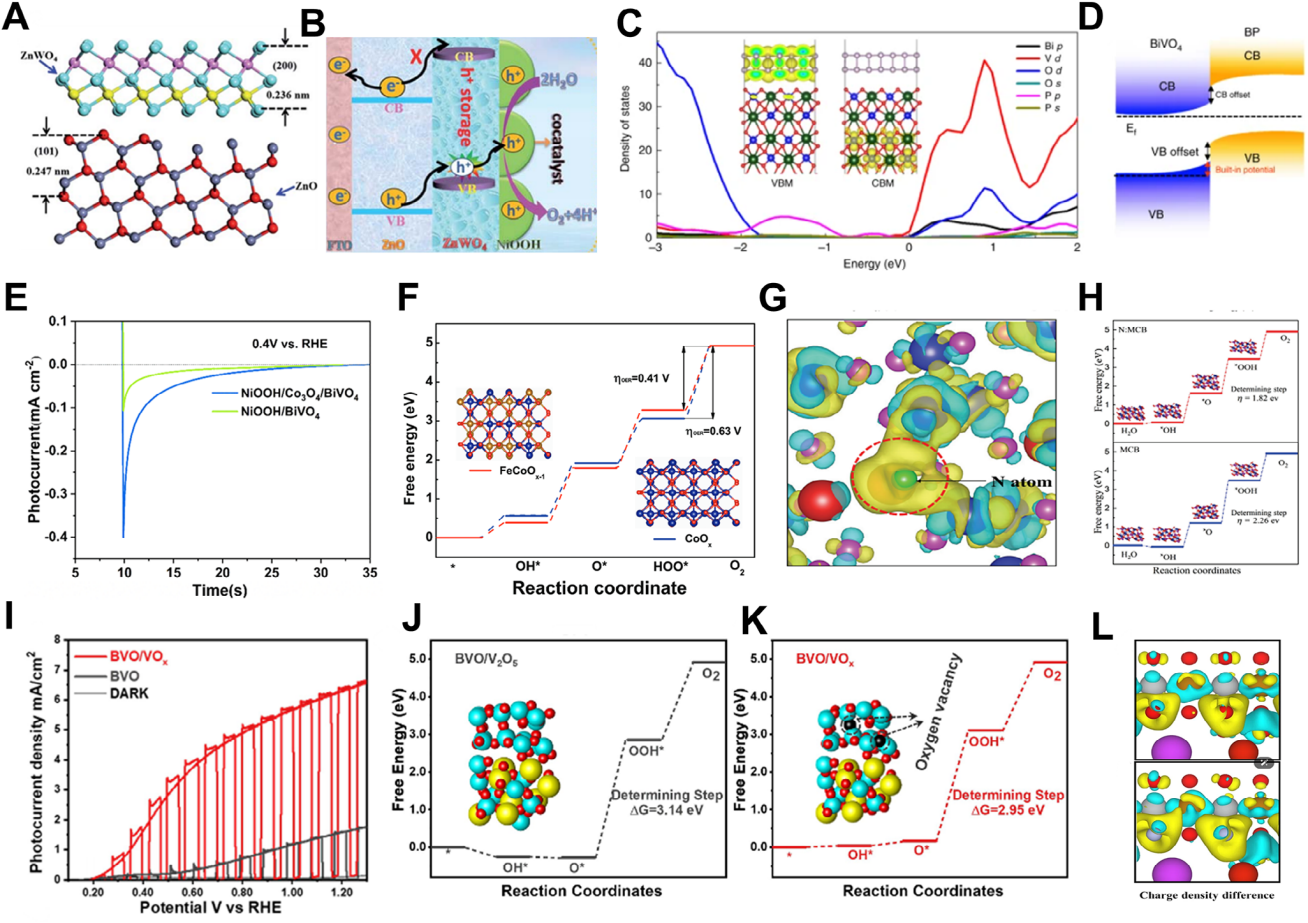
(A) Schematic illustration of the crystal structure of ZnO and ZnWO_4_. (B) Schematic diagram of the charge transport and surface chemical reaction for ZnO/ZnWO_4_/NiOOH [156]. Copyright 2019, Royal Society Chemistry. (C) VB XPS of BiVO_4_ and BP/BiVO_4_ photoanodes. (D) Energy diagram of BP/BiVO_4_ heterojunction [157]. Copyright 2019, Nature Publishing Group. (E) Delay of the cathodic photocurrent curves measured at 0.4 V vs RHE [158]. Copyright 2023, American Chemical Society. (F) Gibbs free energy diagram of the CoO_x_ and FeCoO_x‐1_ [162]. Copyright 2018, Wiley. (G) Schematic diagram of charge density difference by comparing MnCoO_x_/BVO and N: MnCoO_x_/BVO, where blue regions represent the depletion of electrons, and yellow regions represent the accumulation of electrons. (H) Energy diagram of MnCoO_x_/BVO and N: MnCoO_x_/BVO [163]. Copyright 2023, Springer. (I) LSV curves of VO_x_/BiVO_4_ and BiVO_4_. Gibbs free energy diagrams of (J) VO_x_/BiVO_4_ and (K) V_2_O_5_/BiVO_4_ [164]. Copyright 2023, Wiley. (L) Schematic of charge density difference of BiVO_4_/NiFeO_x_ (above image) and BiVO_4_/N:NiFeO_x_ (below image) [165]. Copyright 2021, Nature Publishing Group.

The kinetic process mismatch, such as the charge excitation (ps‐ns), charge extraction (ns‐ms), and surface chemical reaction (ms‐s), requires an overpotential that can generate a sufficiently large built‐in electric field to drive the separation of electron–hole pairs at the SCL and restrain the charge recombination [150]. To decrease the energy loss due to a large overpotential on a photoelectrode system, the charge‐extraction layer (or charge‐transfer layer) and co‐catalyst layer were designed on the photoelectrodes to enhance the ability of the spatial charge separation [151], rather than relying on direct semiconductor‐liquid junctions separating the photogenerated charges. For photoanodes participating in the oxygen evolution reaction (OER), the kinetics of hole transfer and separation largely affect the PEC performance [152, 153]. To improve the photogenerated hole kinetics at the electrode surfaces, the hole transfer layer (HTL), such as Co_3_O_4_, ZnWO_4_, quantum dots, and black phosphorene, was loaded on photoanodes [154]. The basic function of HTL is to provide a great hole transfer pathway between the light‐absorbing semiconductor layer and the cocatalyst layer, which is favorable for inhibiting the charge accumulation and balancing carrier transport across different layers. To effectively extract photogenerated holes from the semiconductor layer, previous research has demonstrated that the position of the HOMO of an HTL is related to the hole transport rate. The proximity of the HOMO energy level of an HTL to that of the semiconductor layer can significantly enhance the interfacial hole transfer rate [155]. For example, vertically well‐aligned zinc oxide (ZnO) nanorods have been used as photoanodes due to their excellent electron transport properties and high specific surface area. However, the serious charge recombination and sluggish OER kinetics restricted their further applications. Bi et al. developed a facile epitaxial growth method to load ZnWO_4_ nanoplates on the (002) facet of a ZnO photoanode, minimizing the energy barrier for transferring holes from the semiconductor bulk to the surface. As shown in Figure 8A, the ZnWO_4_ (200) as an HTL was stacked parallel to ZnO (101). More importantly, the interface lattice mismatch of ZnWO_4_/ZnO was less than 4.45% due to the applied epitaxial growth strategy. The mechanism for enhancing the photoconversion efficiency has been presented in Figure 8B. The matched energy bands between the ZnWO_4_ and ZnO effectively facilitate the hole transfer from ZnO to ZnWO_4_. Then, the stored photogenerated holes in ZnWO_4_ (HTL) could be quickly extracted to the surface cocatalysts for highly efficient PEC water oxidation [156].

It is worth noting that the HTL is usually a p‐type semiconductor that can construct a p‐n junction between a photoelectrode and an HTL. For example, black phosphate (BP), a 2D material with a thickness of 2–20 nm, exhibits p‐type semiconductor properties and a mobility of about 1000 cm^2^ V^−1^ s^−1^. Due to the special material properties of 2D BP, the exfoliated BP nanosheets with 100 nm thickness as an HTL were loaded on BiVO_4_ photoanodes to enhance the ability of hole extraction. Firstly, the intimate contact between the BP nanosheets and the BiVO_4_ substrate forms a strong built‐in field from p‐n junctions. Figure 8C clearly displays the VB‐X‐ray photoelectron spectroscopy (XPS) of BiVO_4_ and BP/BiVO_4_ heterointerface. It is obvious that the loaded BP nanosheets profoundly affect the VB electronic structure of BiVO_4_ through the overlap of the O 2p and P 2p orbitals. In addition, charge transfer that occurs between the O atoms and V atoms at the built‐in electric field (p/n junction) might contribute to the upward bending of the VB, because the O 2p and V 3d orbitals consist of the VB of BiVO_4_. As shown in Figure 8D, the band alignment of BiVO_4_ and BP illustrates the built‐in potential and possible band offsets of the BP/BiVO_4_ heterojunction. This matched band structure can effectively facilitate the electron transport from the CB to the counter electrode and the hole transport from the VB to the photoanode surface [157].

Evaluating the capability of the hole storage was also an issue that needed to be solved in the past. At present, transient cathodic current curves under light‐off conditions have been applied to characterize stored holes at the HTL. Our group compared the delay of the cathodic photocurrent curves between NiOOH/BiVO_4_ and NiOOH/Co_3_O_4_/BiVO_4_, in which Co_3_O_4_ NPs are the HTL and NiOOH is the surface co‐catalyst. The NiOOH/Co_3_O_4_/BiVO_4_ exhibits a higher transient cathodic current density in comparison to NiOOH/BiVO_4_, which can be attributed to the stronger built‐in electric field (Figure 8E). The photocurrent drop from the transient state to the steady state implies the storage ability of photogenerated holes. In comparison, the NiOOH/Co_3_O_4_/BiVO_4_ photoanode has more photogenerated holes than its NiOOH/BiVO_4_ counterpart, further illustrating that the separated holes migrated from the BiVO_4_ bulk are not involved in water oxidation, but are stored in the HTL to improve the hole extraction [158].

#### 4.2.3 Co‐Catalyst Layers

Except for the charge‐extraction layer that can form an interfacial electric field to promote the separation of photogenerated electron–hole pairs, the co‐catalyst layer could also provide a stronger driving force to separate charges and suppress charge recombination. Additionally, the co‐catalyst layer, including metal oxides, metal phosphides, and nonmetallic compounds [159, 160, 161], not only suppresses surface recombination but also enhances the water oxidation activity and water oxidation stability. Our group designed a variety of co‐catalysts decorated on photoanodes to improve charge transfer and extraction. A new type of low‐cost iron‐cobalt oxide (FeCoO_x_) was rationally designed and deposited on BiVO_4_. The prepared FeCoO_x_/BiVO_4_ exhibits an excellent charge separation efficiency of over 90% with a stability of 10 h, implying an outstanding PEC performance. Figure 8F illustrates the free energy diagram of the CoO_x_ and FeCoO_x‐1_ through DFT calculations. The Gibbs free energy barrier of the rate‐determining step for FeO_x‐1_ is lower than its CoO_x_ counterpart. The calculated results reveal that the incorporation of Fe atoms into CoO_x_ generates abundant oxygen vacancies and constructs a p‐n heterojunction promoting the charge transport and extraction [162]. It can be observed that the photoanodes with enough oxygen vacancies display an excellent charge separation efficiency, but the stability of the photoanodes remains a serious issue.

Recently, our group proposed a new heteroatom filling strategy, where the nitrogen atoms were introduced into the MnCoO_x_ co‐catalyst with enriched oxygen vacancies. The optimized N‐filled MnCo_2_O_x_/BiVO_4_ photoanode demonstrates a photocurrent density of 6.5 mA·cm^−2^ at 1.23 *V*_RHE_ under AM 1.5 G illumination, and an excellent stability of over 150 h. A schematic diagram of charge density difference clearly illustrates the effect of N atoms filling on the surface electron structure (Figure 8G). The electron densities around the N atoms have a significant enhancement (yellow region) due to the electron transfer from the Mn atoms to the N atoms, which effectively boosts the capability of hole extraction and transfer. Further DFT calculations (Figure 8H) indicate that the barrier energy of MnCo_2_O_x_ is obviously higher than the N atoms filling MnCo_2_O_x_, implying that the incorporation of N atoms into MnCo_2_O_x_ efficiently improves water oxidation kinetics of the MnCo_2_O_x_/BiVO_4_ photoanode [163].

Non‐noble metal oxides as co‐catalyst layers to be deposited on photoelectrodes typically include iron (Fe), cobalt (Co), nickel (Ni), and manganese (Mn). However, the crystal structure of these metal materials could not perfectly match the crystal structure of semiconductors. To address this issue, our group designed a new type of heterojunction, VO_x_/BiVO_4_, which exhibited a favorable interface charge transfer and surface water oxidation activity. As shown in Figure 8I, the optimized VO_x_/BiVO_4_ demonstrates a photocurrent density of 6.29 mA·cm^−2^ at 1.23 V_RHE_ under AM 1.5 G illumination, corresponding to 385% as high as that of its pristine counterpart. The outstanding water oxidation kinetics can be attributed to the oxygen vacancies in VO_x_, which provide enough undercoordinated sites to strengthen the adsorption of water molecules on the active sites. The Gibbs free energy diagrams of V_2_O_5_/BiVO_4_ and VO_x_/BiVO_4_ clearly prove this viewpoint. In Figure 8J,K, the energy barrier value of the rate‐determining step in VO_x_/BiVO_4_ (2.95 eV) is higher than that of its VO_x_/BiVO_4_ counterpart, indicating a lower energy that drives water oxidation for the VO_x_/BiVO_4_ photoanode [164].

Strengthening the surface charge separation and charge transfer by forming a built‐in electric field has been considered an effective method. However, the relationship between microscopic structures and the capability of charge extraction remains unclear. Bi and co‐workers systematically investigated the effects of low electronegative N atoms on the surface chemical states and electronic structures of BiVO_4_/NiFeO_x_ through XPS and DFT calculations. Figure 8L represents the charge density difference of BiVO_4_/NiFeO_x_ (above image) and BiVO_4_/N:NiFeO_x_ (below image), in which great amounts of electrons are enriched around the Fe sites and Ni sites due to the incorporation of N atoms into NiFeO_x_. Detailed analysis reveals that the V sites of BiVO_4_ receive the electrons from Ni sites for suppressing the dissolution of V^5+^, while electron‐rich Fe sites effectively enhance the separation of the photogenerated electron–hole pairs by improving the hole‐extraction ability [165].

### 4.3 Magnetic Energy

Under the irradiation of solar light, the electrons in the VB are excited toward the CB. Then, the electrons in CB and the left holes in VB serve as reducers and oxidizers to participate in the reduction and oxidation reactions, respectively. However, lacking enough driving force to separate the photogenerated electron–hole pairs limits further applications of photocatalysis [166]. To increase the efficiency of carrier separation and transfer, an external magnetic field as an additional driving force is introduced into a photocatalytic system, thus effectively inhibiting the recombination of carriers and improving photocatalytic activities [102]. Here, we will introduce three mechanisms that explain improved photocatalytic performance caused by a magnetic field: (1) MR effect; (2) Lorentz force; and (3) spin polarization effect.

Firstly, MR, a phenomenon causing resistance to increase or decrease while conductors or semiconductors are placed in a magnetic field, can be categorized as positive and negative. When the MR is negative, a fast charge transfer in photocatalysts can be obtained due to the decreased resistance of semiconductor materials, leading to enhanced photocatalytic activity. The microscopic principle of MR can be explained through some theories, such as the Kondo effect, spin glasses, and the Weyl effect [167, 168]. For example, disordered Mn‐doped Mn_x_VAl_3_ exhibited a logarithmic increase of electrical resistivity, magnetic susceptibility, and specific heat divided by temperature at low temperature (40 K), which can be called the Kondo effect [169]. Although these theories mentioned above have been well developed, the practical applications based on the negative MR effect are rarely reported due to the lack of MR materials. Hence, the negative MR effect is a promising mechanism waiting for further development. It is believed that once some photocatalysts with a high solar‐to‐energy conversion efficiency have a MR effect by regulating crystal structures and electronic structure, the photocatalytic performance of these common photocatalysts can be further enhanced.

The second mechanism is the Lorentz force. It is well known that different charged molecules (positively or negatively charged) in an external field usually have opposite movement directions. This spatial separation of different charged molecules is favorable for some photocatalytic reactions, in which the agglomeration of molecules and recombination of charges can be inhibited due to different migration directions. A formula is given to calculate the Lorentz force and its direction when a charged particle moves in an external magnetic field [170, 171].

(5) $$\;F_{\mathrm{Lorentz}}=q\vec{v}\times \vec{B}$$

where *q* is the charge of particles, *v* is the velocity, and *B* is the external magnetic field intensity. Since photogenerated electrons and holes possess opposite, the Lorentz forces acting on them are oriented in opposite directions, thereby promoting spatial electron‐hole separation. On the other side, the Lorentz force can also produce an internal electric field and speed up ion transfer in solution. Specifically, the holes and electrons enforced by reverse Lorentz force are intensely packed, respectively, thereby forming an interfacial built‐in electric field with opposite charges, which further separates holes and electrons through electrostatic force. Through X‐ray absorption spectroscopy and DFT calculations, Chen and coworkers revealed that Lorentz force induced by defective structures can regulate electronic and magnetic properties of semiconductor materials, thereby suppressing the recombination of photogenerated carriers [172].

The third mechanism is spin polarization. It is well known that electron migration is a vital process in photocatalysis, and spin is one of the intrinsic features of electron itself [173]. Generally, electron spin can be categorized into two types, which are “spin up” and “spin down”, respectively. Since the electron spin can be controlled and regulated by an external magnetic field, we can use a magnetic field to directly manipulate the photocatalysis process. When electrons in a particle are aligned in a particular direction, rather than being random or balanced between the “up” and “down” spin states, this degree of directional arrangement is denoted as spin polarization. To quantify the degree of spin polarization, we use the formula *I*_Spin polarization_ = (*n*_up_ − *n*_down_)/(*n*_up_ + *n*_down_) to characterize the anisotropy of spin polarization, in which *n*_up_ and *n*_down_ refer to the spin‐up state and spin‐down state, respectively [49]. Spin polarization can significantly affect the photocatalysis process, especially in materials where the electron spin influences reaction kinetics and charge transfer.

In a photocatalysis process, electrons are excited from VB to CB, generating photoinduced electron–hole pairs that participate in redox reactions. The spin polarization impacts this process in a few ways. Firstly, spin‐polarized photocatalysis materials can efficiently facilitate the separation of photoexcited electrons and holes. Spin–orbit coupling (SOC), an interaction between electron spin and motion (orbital angular momentum), is crucial in many areas of physics and materials science due to its significant effects on electronic, magnetic, and optical properties of semiconductor materials. For a photocatalytic material with a strong SOC, great amounts of photoexcited electrons in CB would undergo a spin flip, which makes it impossible for electrons to recombine with unflipped photoexcited holes in VB unless holes also undergo the same spin flip. Improved charge separation enhanced the lifetime of carriers, making them more available to drive redox reactions. The second important effect is selective reaction pathways. Certain reactions, such as water oxidation, pollutant degradation, and CO_2_ reduction, require electrons and intermediates with specific spin orientations [104, 174]. Photocatalysts with a strong polarization can promote specific spin states in reaction intermediates, optimizing the reaction pathways with a lower Gibbs free energy.

Previous reports have shown that the application of an external magnetic field in the field of catalysis is a promising strategy. By regulating the direction and intensity of the external magnetic field, a higher photocatalytic activity and product selectivity are achieved compared with those without an external magnetic field. Although excellent photocatalytic performance with the assistance of a magnetic field has been achieved in the past two decades, the understanding of how an external magnetic field influences photocatalysis remains vague due to the limitations of the available equipment and the incompleteness of the underlying theories. In order to gain further insight into magnetic field‐assisted photocatalysis, this section will present examples of the latest achievements from a physical perspective, which can facilitate a more comprehensive understanding of the mechanisms of external magnetic field‐assisted photocatalysis.

#### 4.3.1 Magnetoresistance Effect

MR effect is a vital mechanism in magnetic‐assisted photocatalysis. We can use the equation MR% = (*R*_H_ − *R*_0_)/*R*_0_ to accurately quantify the effect of external magnetic field on MR of materials, in which *R*_H_ and *R*_0_ are the resistances with and without an external magnetic field, respectively [97]. In the presence of an external magnetic field, the semiconductor materials with a negative MR can result in reduced resistance in a process of charge transfer, thereby facilitating the spatial separation of photogenerated electron–hole pairs. Effective charge separation makes the carriers survive for a longer time, leading to faster chemical reaction kinetics [175]. On the other hand, charge mobility, determining how quickly and efficiently charge carriers travel to the surface reaction sites, can also be influenced by the MR effect. For example, photocatalysts with positive MR have a smaller charge mobility in comparison to a photocatalyst with a negative MR. Li and coworkers successfully fabricated an α‐Fe_2_O_3_/reduced graphene oxide hybrid nanostructure (α‐Fe_2_O_3_/rGO) photocatalyst through a facile hydrothermal reaction. The charge transfer efficiency of α‐Fe_2_O_3_/rGO has been significantly enhanced under an external magnetic field, which can be attributed to a negative MR effect.

**FIGURE 9:**
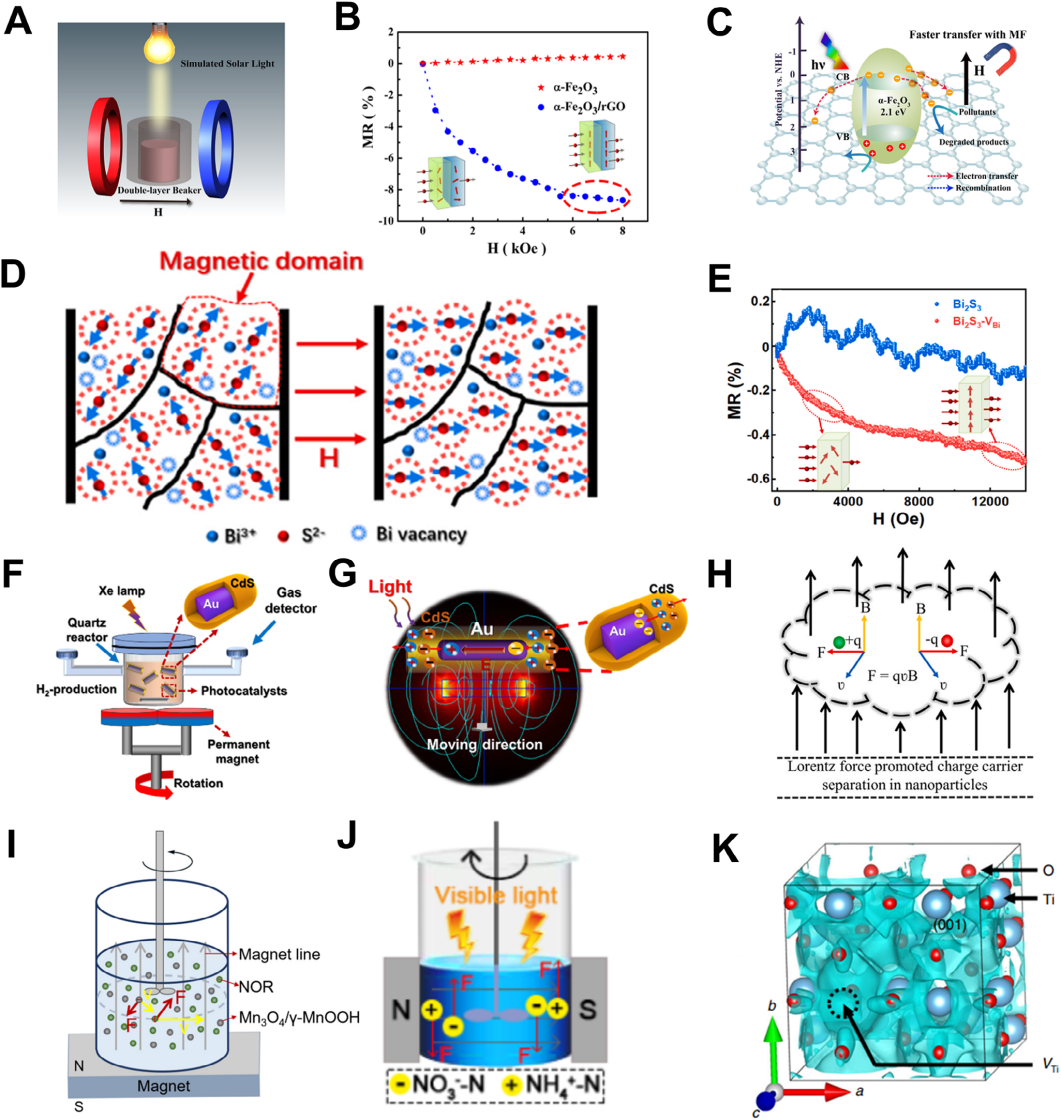
(A) Schematic diagram of magnetic‐assisted photocatalysis setup. (B) Magnetoresistance (MR) curves with various magnetic intensities from 0 kOe to 8 kOe for α‐Fe_2_O_3_ and α‐Fe_2_O_3_/Rgo. (C) Schematic illustration of proposed photocatalysis mechanism in α‐Fe_2_O_3_/rGO with an external magnetic field [176]. Copyright 2018, American Chemical Society. (D) Schematic diagram of electron spin state with and without a magnetic field. (E) Magnetoresistance (MR) curves with various magnetic intensities from 0 kOe to 12 kOe for Bi_2_S_3_ and Bi_2_S_3_‐V_Bi_ at room temperature [178]. Copyright 2023, Elsevier. (F) Schematic diagram of a photocatalysis setup for producing hydrogen under an external magnetic field. (G) Schematic diagram of the influence of a magnetic field on the moving Au NR‐CdS NP core‐shell nanostructure [179]. Copyright 2020, Elsevier. (H) Two opposite Lorentz forces are exerted on two molecules with opposite electronegativity under an external magnetic field [170]. Copyright 2022, Elsevier. (I) Schematic illustration of the positive effect on norfloxacin degradation [181]. Copyright 2021, Elsevier. (J) Schematic illustration of different movement directions on NO_3_^−^‐N and NH_4_^+^‐N ions under a permanent magnet [182]. Copyright 2018, Elsevier. (K) 3D spatial distributions of spin polarization on Ti‐defected TiO_2_ photocatalysts [103]. Copyright 2020, Nature Publishing Group.

Figure 9A exhibits a schematic diagram of the electromagnet‐photocatalysis setup, where an electromagnet with a magnetic field ranging from 0 to 10 kOe and a quartz double‐layer beaker as the photocatalytic reactor is applied. When the external magnetic field varies from 0 to 8 kOe, the MR% of α‐Fe_2_O_3_/rGO photocatalysts exhibits an obvious negative MR effect with a maximum value of about 9% at 8 kOe. On the contrary, there are no obvious negative effects on pure α‐Fe_2_O_3_, confirming that the MR effect is markedly evident in α‐Fe_2_O_3_/rGO, instead of pure α‐Fe_2_O_3_ (Figure 9B). This negative MR effect has a positive effect on organic pollutant degradation. The prepared α‐Fe_2_O_3_/rGO photocatalyst was applied to degrade rhodamine B dye (RhB) under an external magnetic field with different intensities. In an external magnetic field of 8 kOe, approximately 85% of RhB is degraded within 40 min, which is evidently higher than its counterpart in an external magnetic field of 0 kOe (50%). The improved degradation performance can be attributed to a fast electron transfer due to the negative MR effect. In detail, carriers can quickly transfer from α‐Fe_2_O_3_ to rGO by imposing an external magnetic field. As a result, more charge carriers can transfer to the active sites and participate in photocatalytic degradation (PD) (Figure 9C) [176].

A similar MR effect enhancing the photocatalytic activity was observed in a Z‐scheme ZnFe_2_O_4_/AgBr magnetic photocatalyst. As a p‐type semiconductor, ferromagnetic nanosized ZnFe_2_O_4_ has a negative MR, enabling it to be controlled by an external magnetic field. A low VB of the surface AgBr further enhances the photocatalytic efficiency due to an improved electron transfer. Under a magnetic field of 1000 Oe, the photocurrent density of ZnFe_2_O_4_/AgBr is 151.8 µA/cm^2^, which is obviously higher than that of ZnFe_2_O_4_/AgBr without an external magnetic field (75.1 µA/cm^2^). The significantly enhanced photocatalytic performance under an external magnetic field is mainly ascribable to an increase in the MR effect. As the intensity of a magnetic field increases from 2 to 8 kOe, the value of the negative MR% on the ZnFe_2_O_4_/AgBr composites also rises from 6% to 11% [177].

Although loading magnetic materials on conventional photocatalysts is a promising strategy to take advantage of the magnetic energy of an external magnetic field, a variety of pure semiconductor photocatalysts cannot fully utilize magnetic energy due to the lack of the MR effect. Therefore, developing conventional photocatalysts with an excellent MR effect is challenging and promising. Recently, a ferromagnetic Bi_2_S_3_ was prepared by the introduction of a Bi vacancy in the Bi_2_S_3_ lattice. The magnetism properties of Bi_2_S_3_‐V_Bi_ mainly originate from the spin polarization of the 2p‐orbital electrons in S atoms, and S atoms around the Bi vacancies construct its magnetic moment simultaneously. Under an external magnetic field, a negative MR effect occurs in Bi_2_S_3_‐V_Bi_ photocatalysts due to the tunneling of spin‐polarized electrons in the S‐2p orbital. As illustrated in Figure 9D, a spin polarization process can be observed on Bi_2_S_3_‐V_Bi_ photocatalysts with a gradually increased magnetic intensity, in which the magnetic torque of atoms in different magnetic domains tends to arrange in the same direction. As the intensity of a magnetic field rises from 0 to 1.2 kOe, a negative MR% of Bi_2_S_3_‐V_Bi_ reaches around 0.5%, obviously higher than its pristine Bi_2_S_3_ counterpart (0.1%) (Figure 9E). This enhanced negative MR effect contributes to an improved interfacial charge transfer and inhibits the recombination of photogenerated electron–hole pairs, thereby leading to an outstanding hydrogen evolution efficiency [178].

#### 4.3.2 Lorentz Force

Due to the insufficient charge separation and poor conductivity of photocatalysts, a recombination of photogenerated electron–hole pairs would occur in both the bulk and surface of semiconductors, resulting in a reduced photocatalytic efficiency. In this case, the Lorentz force, an acting force applied to moving charges in an external magnetic field, was widely and frequently utilized to improve the photocatalytic performance. Two main mechanisms explain the reason for the improved photocatalytic efficiency: (1) suppressing recombination of photogenerated electron–hole pairs, and (2) regulating the motion of photocatalysts and reactant molecules.

A Lorentz force‐assisted photocatalytic setup commonly is composed of three sections (Figure 9F). A xenon lamp is the simulated sunlight. A quartz reactor is applied as a reaction container. Under the reactor, a constantly rotating magnet can provide an external magnetic field with a variable intensity. A Lorentz force caused by a permanent magnet can efficiently suppress the recombination of electrons and holes during photocatalysis. Gao and coworkers designed a metal‐semiconductor core‐shell hybrid nanostructure, where CdS NPs were loaded on gold nanorods (Au NR). Under an external magnetic field, the highest electromotive force is formed in the middle of the Au NR, and gradually decreases for both sides (Figure 9G), thereby constructing a homogeneous and parallel electric field. Due to the electrostatic interaction, accumulated charges at the ends of Au NR can generate an electric field inside the Au NR near the surface, which can drive the charge separation in the surface‐loaded CdS NPs. With the assistance of the Lorentz force, the photocatalytic efficiency of hydrogen production can be increased to around 110% [179].

Similarly, Ni_x_Mn_(0.5‐x)_O as a hierarchical catalyst was assembled on magnetic nanoparticles (MNPs) to construct a core/shell NP. The moved charges in MNPs@ Ni_x_Mn_(0.5‐x)_O photocatalysts experience a Lorentz force perpendicular to the plane between the direction of motion and the magnetic field. As shown in Figure 9H, photogenerated electrons and holes migrate in opposite directions by the opposite Lorentz force, which efficiently promotes the separation of charges in NPs. Due to an excellent charge separation efficiency under Lorentz force, 98% of sulfamethoxazole was degraded in the MNPs@ Ni_x_Mn_(0.5‐x)_O@MNPs photocatalysts within 30 min, which is obviously higher than its counterpart without a magnetic field (65% degradation) [170].

It is well known that loading the electron extraction layer on the surface of photocatalysts is a promising strategy to boost the photocatalytic performance of the hydrogen evolution reaction. A challenging issue is how to attract more electrons aggregating on the surface of the electron extraction layer. The introduction of the Lorentz force can efficiently solve this issue. For example, Ag NPs can act as electron traps to increase the charge transfer ability of Ag@g‐C_3_N_4_ photocatalysts. Under an external magnetic field, more photogenerated electrons aggregate in the Ag NPs, leading to a significantly enhanced charge separation efficiency. The improved charge separation can be attributed to the Lorentz force, which makes electrons with negative charges transfer from the CB to the surface Ag NPs [180].

Generally, the agglomeration of photocatalysts in aqueous solution possibly decreases the reaction performance due to a reduced number of active sites. The Lorentz force endowed by an external magnetic field can efficiently suppress this phenomenon. For instance, the Mn_3_O_4_/γ‐MnOOH as photocatalysts with negative charges and norfloxacin (NOR) as a reaction substance with positive charges usually have opposite Lorentz forces according to the Lorentz force rule. Therefore, the movement direction of Mn_3_O_4_/γ‐MnOOH is outward. On the contrary, the movement direction of NOR is inward (Figure 9I), which significantly improves a uniform distribution of catalysts and reactant molecules [181]. Owing to an opposite movement direction for molecules with opposite electronegativity under the Lorentz force, some difficult chemical reactions in the past can be achieved, including the denitrification of nitrate (NO_3_^−^‐N) and ammonia (NH_4_^+^‐N) simultaneously. Under a 3D/2D Mn_2_O_3_/g‐C_3_N_4_ photocatalytic system, a high removal efficiency of 94.5% and 97.4% for NO_3_^−^‐N and NH_4_^+^‐N can be obtained after imposing a magnetic field. Lorentz force plays an important role in enhancing the absorption ability of NO_3_^−^‐N and NH_4_^+^‐N on the surface of 3D/2D Mn_2_O_3_/g‐C_3_N_4_ photocatalysts.

As illustrated in Figure 9J, negatively charged NO_3_^−^‐N molecules experience a repulsive force, while positively charged NH_4_^+^‐N molecules experience an attractive force due to the negatively charged 3D/2D Mn_2_O_3_/g‐C_3_N_4_ photocatalysts. Upon introducing an external magnetic field in this photocatalytic system, the Lorentz force is pointed toward or deviated from the solution surface according to the different electronegativity of charged molecules. Therefore, NO_3_^−^‐N and NH_4_^+^‐N will migrate to opposite directions under the influence of the Lorentz force. Consequently, the Lorentz force makes it possible for negatively charged NO_3_^−^‐N and positively charged NH_4_^+^‐N to approach the active sites on the Mn_2_O_3_/g‐C_3_N_4_ photocatalysts, leading to an improved molecule absorption and an outstanding photocatalytic performance for the concurrent redox denitrification [182].

#### 4.3.3 Spin Polarization

Spin polarization is an interesting physical phenomenon that is widely utilized to control the electronic spin states associated with photocatalytic reactions. Recent reports have shown that photocatalytic performance can be enhanced by modulating the electron spin states. Firstly, spin polarization can extend the lifetime of photogenerated carriers, which is of great importance for improving the charge transfer efficiency. Secondly, spin polarization can lower the reaction barrier and influence reaction pathways, thereby enhancing product selectivity for some value‐added reactions, such as CO_2_ reduction [183]. Spin polarization can commonly be obtained by a magnetic field. Briefly, the interaction of electron spin–orbit and magnetic field results in the ordered arrangement of the electron spin, forming a magnetic moment. Under the influence of an external magnetic field, the orientation of electron spin is manipulated, thereby achieving a spin polarization effect. For example, ferromagnetic ZnFe_2_O_4_ (ZFO) as photoanodes was applied to participate in the water oxidation reaction. The ferromagnetic property was enhanced by introducing cation disorder and oxygen vacancies. The optimized ZFO photoanodes exhibit a higher photocurrent density in comparison to the untreated ZFO photoanodes. The significantly improved photocatalytic performance can be attributed to spin polarization caused by an external permanent magnet, which efficiently inhibits charge recombination and boosts the interfacial charge transfer. In detail, the spin states of photoinduced electrons and holes are opposite to each other without the spin polarization. Based on the theory of hyperfine interaction and SOC, the recombination of electrons and holes can occur simultaneously. However, with the assistance of an external magnetic field, the same spin state of excited electrons and holes can be obtained, resulting in an inhibited recombination of the excited electrons and holes [184].

Similar to ZnFe_2_O_4_, the spin polarization of delafossite AgFeO_2_ can be enhanced by an external magnetic field. Dong et al. designed three different phases of AgFeO_2_, including the 3R phase, 2H phase, and 3R‐2H mixed phase. Theoretical calculations reveal significantly different spin polarization and ferromagnetic properties for different crystal phases. Prepared AgFeO_2_ photocatalysts with the 3R phases exhibit the largest spin polarization intensity, which is the reason for the improved photocatalytic performance for hydrogen production. The spin density distribution of different crystal phases clearly illustrates that the O atoms and Ag atoms have the same spin density distribution, and the corresponding values are relatively small for different crystal phases. However, the spin density of the Fe atoms has an obvious difference. Firstly, the spin intensity of Fe is greatly higher than that around the O atoms and Ag atoms. Secondly, the spin‐up and spin‐down of the Fe atoms in the 3R phase counteract each other, and a net magnetic moment occurs around the 3R phase. In contrast, there is no net magnetic moment in Fe atoms of the 2H phase, confirming that AgFeO_2_ with the 3R phase has a stronger spin polarization effect. The electronic structure of AgFeO_2_ further demonstrates the importance of Fe atoms in the 3R phase for the spin polarization effect, in which the spin‐up state and spin‐down state of the Fe‐3d states occupy the VB from −10 to −7 eV and 1 to 5 eV, respectively [185].

Although spin polarization can significantly enhance the photocatalytic performance in theory, few works have been reported because a variety of highly efficient semiconductor photocatalysts are non‐magnetic. To apply the spin polarization effect in photocatalysts without the ferromagnetism property, defect engineering was utilized to manipulate their electron spin polarization. Various concentrations of Ti vacancies were introduced in TiO_2_ photocatalysts, thereby obtaining an optimized Ti_0.936_O_2_ photocatalyst with a spin polarization effect. The as‐prepared Ti_0.936_O_2_ exhibited excellent charge separation efficiency and surface reaction kinetics due to the high degree of spatial spin polarization. According to the theoretical calculations for the electronic structure of Ti‐defected TiO_2_, the spin‐up state of electrons in Ti‐defected TiO_2_ is obviously lower than that of the electron spin‐down state, leading to more spin‐down photogenerated electrons under light irradiation. As shown in Figure 9K, 3D spatial distributions of spin polarization illustrate that negative spatial spin polarization occupies a large volume of the supercell, indicating little possibility of spin polarization reversal in real space. At a magnetic field of 8000 Oe, the Rh B degradation efficiency in Ti_0.936_O_2_ photocatalysts was increased by 54% in comparison to that without an external magnetic field, which can be attributed to the inhibition of combination between the hydroxyl radicals with anti‐parallel spin directions [103].

Except for constructing metal defects in photocatalysts, doping magnetic ions in commonly highly efficient photocatalysts can also endow photocatalysts with a spin polarization property. Gao and coworkers successfully doped magnetic Ni elements into a CdS/MoS_2_ heterostructure. The spin polarization effect can be detected in both CdS and MoS_2_ of the CdS/MoS_2_ photocatalysts. The hydrogen evolution efficiency of Ni‐doped CdS/MoS_2_ is 2.64 times higher than that of the undoped CdS/MoS_2_ photocatalysts. Furthermore, with the application of an external magnetic field, the hydrogen evolution performance is increased to 1.47‐fold that of Ni‐doped CdS/MoS_2_ without a magnetic field. Theoretical calculations further reveal that the improved photocatalytic performance is assigned to the spin polarization caused by the Ni‐doping strategy and the parallel arrangement of the electron state, which facilitates the charge separation efficiency and surface reaction kinetics [186].

### 4.4 Mechanical energy

Piezocatalysis is gaining wide interest in sustainable energy conversion [187]. It offers a novel strategy to harvest mechanical energy for driving a variety of chemical reactions. When mechanical stress and pressure are applied to piezoelectrics, a localized electric field and surface charges can be generated on piezoelectrics in response to the mechanical deformation. These charges can interact with reactant molecules, thereby driving chemical reactions. In addition, surface charges can generate reactive species (i.e., hydroxyl radical (·OH) and peroxyl ion (·O_2_^−^)), promoting chemical transformations [188]. Recently, the combination of the piezoelectric effect on photocatalysis has shown a promising prospect. Firstly, the most important effect of the piezoelectric effect is to promote the separation and transfer efficiency of photogenerated electrons and holes. Owing to mechanical deformation caused by an external strain, stress‐induced charges can generate an internal electric field, effectively reducing the recombination of photogenerated carriers. Secondly, the piezoelectric effect promotes the formation of ·OH and ·O_2_^−^, which are crucial for photocatalytic reactions, such as pollutant degradation and water splitting reactions. Thirdly, some piezoelectric materials can modify the local electronic structure and band position of the photocatalyst, possibly broadening its light absorption range [189].

In the following section, we will introduce some classic single‐component piezoelectric semiconductors, including ternary metal oxides (i.e., BaTiO_3_, ZnSnO_3_) and binary metal compounds (i.e., CdS, ZnO) as photocatalysts to participate in chemical reactions [190]. Composite photocatalysts consisting of piezoelectric materials and semiconductors are also involved. Moreover, different strategies introducing an external pressure, such as high‐frequency ultrasonic vibration and low‐frequency shear force, are also included.

#### 4.4.1 Ternary Metal Oxide

**FIGURE 10:**
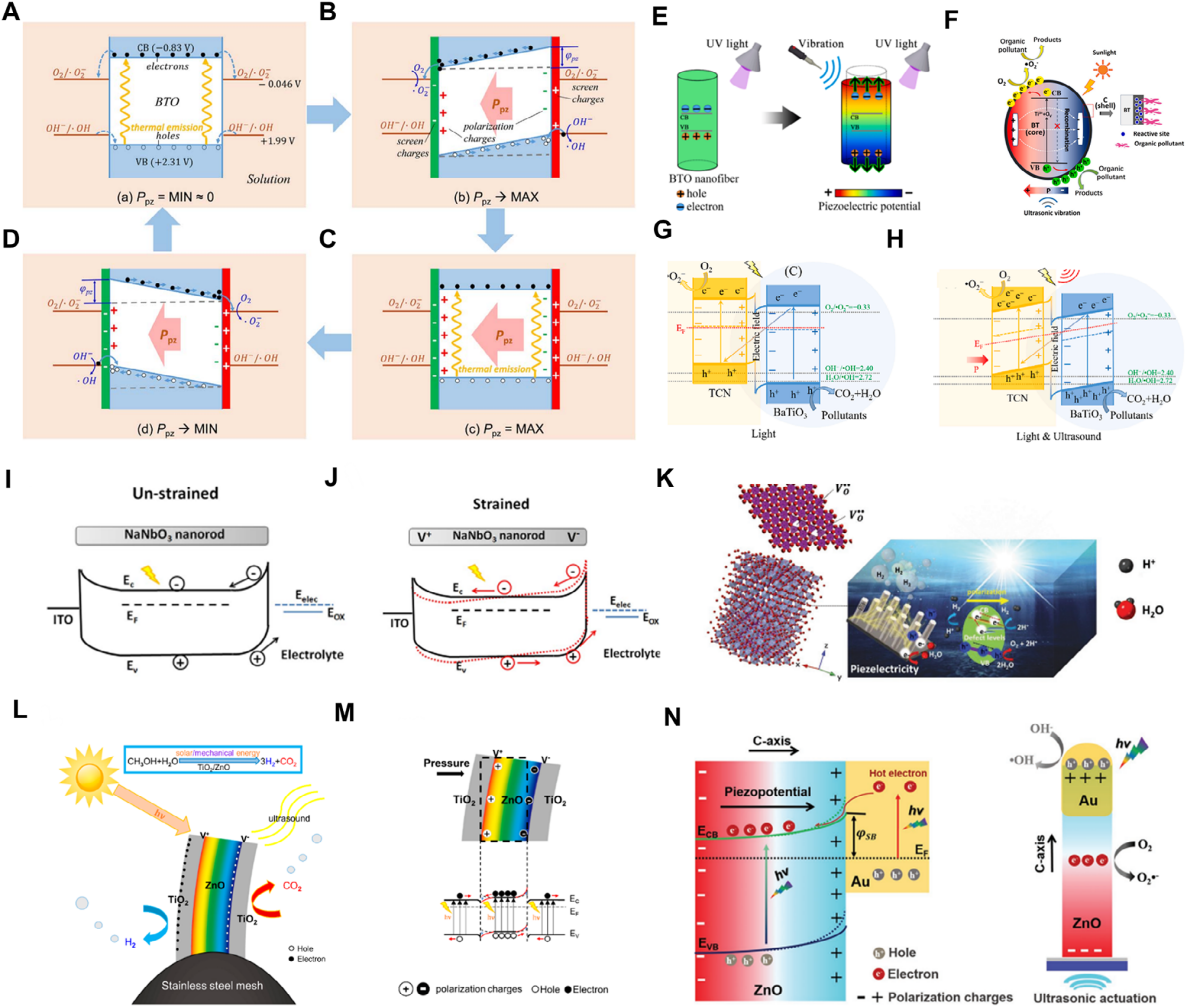
(A–D) Principal diagrams of piezo‐photocatalysis composed of band structure and surface reaction during the ultrasonic vibration: (A) piezoelectric potential equals 0; (B) piezoelectric potential tends to maximum; (C) piezoelectric potential equals maximum; (D) piezoelectric potential tends to minimum [192]. Copyright 2018, Elsevier. (E) Schematic diagram of piezo‐photocatalysis in BaTiO_3_ nanofibers [193]. Copyright 2024, Elsevier. (F) Schematic mechanism of degrading organic pollutants in BaTiO_3_@C [194]. Copyright 2024, Elsevier. (G) Energy band diagram of BaTiO_3_/g‐C_3_N_4_ under light illumination. (H) Energy band diagram of BaTiO_3_/g‐C_3_N_4_ under light illumination and ultrasonic vibration [195]. Copyright 2024, Elsevier. (I) Band alignment of NaNbO_3_ nanorods without piezoelectric potential. (J) Band alignment of NaNbO_3_ nanorods with piezoelectric potential [198]. Copyright 2017, Elsevier. (K) Working mechanism of the HER via the synergistically piezo‐photocatalytic effect of ZnSnO_3_ NWs with oxygen vacancies [196]. Copyright 2019, Wiley. (L) Piezo‐photocatalytic mechanism of mesh‐based TiO_2_/ZnO nanowires under solar and ultrasonic irradiation. (M) Energy band diagram of TiO_2_/ZnO nanowire under the synergistic influence of solar and ultrasonic irradiation [203]. Copyright 2018, American Chemical Society. (N) Schematic illustration of the improved photocatalytic performance induced by both the piezoelectric effect and unique asymmetric nanostructure under light irradiation and ultrasonic actuation [202]. Copyright 2020, Wiley.

BaTiO_3_, a piezoelectric material that can generate a high piezoelectric potential, has been utilized in the field of piezocatalysis and piezo‐photocatalysis. Many approaches can generate an external strain on piezoelectric materials. The most common strategy is to apply a high‐frequency ultrasonic vibration, which deforms piezoelectric materials effectively due to the extreme acoustic pressure of ultrasonic waves and the local high pressure by the collapse of active bubbles [191]. The effect of high‐frequency strain on surface redox reactions and band structures is shown in Figure 10A–D. There is no piezoelectric potential within BaTiO_3_ in the absence of external mechanical energy (Figure 10A). When a piezoelectric polarization is introduced by external strain, the free electrons and holes are attracted into an opposite direction due to the formed built‐in electric field (Figure 10B), leading to a tilted CB and VB. Moreover, the larger piezoelectric potential contributes to a fast chemical reaction of holes and electrons with the dissolved oxygen and hydroxyl to form radicals. However, the piezoelectric potential decreases to zero due to the new balance between polarization charges and screen charges when external pressure retains the maximum (Figure 10C). This potential equilibrium will be broken with falling pressure, resulting in the reverse charge transfer and redox reactions (Figure 10D) [192].

On the basis of piezoelectric potential improving the immigration of electrons and holes, BaTiO_3,_ as a piezoelectric semiconductor, was applied in photocatalysis. For example, Wu and coworkers successfully prepared BaTiO_3_ nanofibers by a feasible hydrothermal synthesis method for the degradation of organic pollutants. With the UV‐light irradiation and high‐frequency vibration simultaneously, the piezo‐photocatalysis performance is obviously higher than the photocatalysis performance, which can be attributed to the piezoelectric potential formed by external mechanical energy. In detail, a serious recombination of photogenerated electrons and holes occurs in BaTiO_3_ photocatalysts under UV light. After introducing a high‐frequency vibration on BaTiO_3_, the recombination of carriers is greatly restrained due to the piezoelectric‐induced potential, which is favorable for dye decomposition (Figure 10E) [193].

Recently, a variety of BiTiO_3_‐based heterostructures have been designed to further improve the photocatalytic performance. It is well known that BaTiO_3_ has poor photocatalytic properties due to its wide bandgap (Eg > 3.2 eV). An effective strategy is to regulate the band structure of BaTiO_3_ by constructing heterostructures. The semiconducting BaTiO_3_@C photocatalyst was prepared by a glucose‐assisted hydrothermal method. Owing to the reducing effects of glucose in a hydrothermal process, the Ti^4+^ on the BaTiO_3_ surface was reduced to Ti^3+^, resulting in the formation of abundant oxygen vacancies. The additional glucose was carbonized to a carbon shell around oxygen vacancies‐enriched BaTiO_3_. In comparison to raw BaTiO_3_, BaTiO_3_@C exhibited a higher photocatalytic efficiency for degrading organic pollutants. The enhanced photocatalytic performance is ascribed to a built‐in electric field caused by the piezoelectric potential, which originated from continuous ultrasonic vibration. The photoinduced electrons and holes migrated in completely opposite directions in an electric field. As a result, the recombination of charge carriers was inhibited and the lifetime of electrons and holes was prolonged (Figure 10F). In addition, the constructed oxygen vacancies and surface carbon shell changed the band arrangement of BaTiO_3_, in which the E_CB_ and E_VB_ of BaTiO_3_@C were more negative than the raw BaTiO_3_, leading to large amounts of ·O_2_^−^ participating in oxidation reactions for degrading organic pollutants [194].

The other effective strategy for fully taking advantage of the piezoelectric effect is to construct two‐layer piezoelectric materials. BaTiO_3_ with the piezoelectric effect was loaded on one‐dimensional tubular g‐C_3_N_4_ with the same piezoelectric effect by a self‐assembly hydrothermal method. The piezo‐photocatalysis efficiency for degradation of tetracycline hydrochloride in BaTiO_3_/g‐C_3_N_4_ composites reached 91.0% within 60 min under visible light and high‐efficiency ultrasonic vibration. In contrast, the degradation rate of the BaTiO_3_/g‐C_3_N_4_ composites under visible light irradiation was 81.8%, illustrating the advantages of dual piezoelectric photocatalysts. The favorable effect of the ultrasonic vibration on the band energy structure is shown in Figures 10G,H. It is obvious that the Fermi level of BaTiO_3_/g‐C_3_N_4_ is flat under visible light illumination. When an external high‐efficiency vibration is applied in the BaTiO_3_/g‐C_3_N_4_ photocatalysts, the Fermi level and corresponding VB/CB are pitched due to the piezoelectric potential, resulting in more photogenerated electrons and holes accumulating on the CB of BaTiO_3_ and VB of g‐C_3_N_4_. Therefore, a higher degradation efficiency on BaTiO_3_/g‐C_3_N_4_ photocatalysts can be achieved [195].

Besides BaTiO_3_, other piezoelectric materials, such as ZnSnO_3_ [196], KNbO_3_ [197], and NaNbO_3_ [198], have also been considered as effective photocatalysts for piezophototronic applications. Khare et al. applied NaNbO_3_ into a particulate suspension for degrading the methylene blue organic dye, and the photoelectrode system for PEC water splitting. Especially for PEC water splitting, the photocurrent density of the NaNbO_3_ photoanodes increased to 1.02 mA/cm^2^ with the assistance of the piezoelectric effect, which is higher than its counterpart without the piezoelectric effect (1.02 mA/cm^2^). The improved water‐splitting performance is mainly due to the inclination of band alignment. In the presence of the high‐efficiency ultrasonic vibration, more photogenerated holes migrate to the surface, and the VB is lifted more upward so that it becomes close to the oxidation potential. Therefore, holes can easily migrate from the bulk to the surface for water oxidation. Meanwhile, lifting of the CB at the electrolyte interface facilitates the electron transfer towards the counter electrode for water reduction (Figure 10I–J) [198].

ZnSnO_3_, a R3c ferroelectric LiNbO_3_‐type piezoelectric material, has a large spontaneous polarization (P) of approximately 50.2 µC cm^−2^ along the z‐axis direction and photo response capability. Hence, ZnSnO_3_ as a piezoelectric material was used to produce hydrogen and degrade organic dyes under the synergistic piezoelectric effect and photoresponse process. To further enhance hydrogen production efficiency, great amounts of oxygen vacancies (Ov) were doped into the ZnSnO_3_ lattices. As a result, the hydrogen production rate of Ov‐ZnSnO_3_ photocatalysts reached 6000 µmol g^−1^ within 7 h, larger than its counterparts without Ov (approximately 2500 µmol g^−1^ within 7 h). Figure 10K clearly reveals how external energy fields (including visible light illumination and pressure) and oxygen defects affect energy band arrangement and charge separation. Through light irradiation, the photogenerated electrons and holes are excited to the CB and VB, respectively. The incorporation of external mechanical energy into the photocatalysis system induces a stress‐induced piezoelectric polarization, which effectively drives the separation of electrons and holes opposite directions, leading to a significantly improved charge transfer capability [196]. Similar to ternary metal oxides, some ternary metal sulfides also exhibited the piezoelectric property. For example, Cd_x_Zn_1‐x_S, as a photocatalytic piezoelectric material, was applied to produce hydrogen peroxide (H_2_O_2_). Benefiting from the nano‐branch structure, mechanical energy can be effectively converted into electric energy under ultrasound conditions, leading to a higher concentration of charge carriers. The optimized Cd_0.5_Zn_0.5_S photocatalyst exhibited the highest performance with a rate of 21.9 µmol g^−1^ h^−1^ without any sacrificial agent [199].

In addition to external mechanical energy forming a polarization field, the introduction of lattice strain into photocatalysts was also a cost‐effective and facile strategy. The lattice strain in Bi_2_MoO_6_ nanosheets can be obtained through synergistic indium doping and the formation of Ov, which engendered tensile strain and compressive strain, respectively. The interaction of these two‐dimensional defects produced one‐dimensional dislocation defects, resulting in significant three‐dimensional lattice distortions. Comprehensive characterizations revealed that the enhancement of local lattice strain effectively strengthened charge carrier separation and the genesis of surface‐active sites. Therefore, the optimized Bi_2_MoO_6_ nanosheets with lattice strains as the photocatalyst exhibited significantly enhanced activity for degrading tetracycline, ciprofloxacin, oxytetracycline, and norfloxacin [200].

#### 4.4.2 Binary Metal Compounds

Although applying ternary metal oxides (including BaTiO_3_, KNbO_3_) as photocatalysts for degrading organic pollutants or producing hydrogen is a promising strategy due to their excellent piezoelectric response, the wide bandgap and mismatched band alignment still limit their photocatalytic efficiency. Binary metal compounds, such as CdS and ZnO, not only have an excellent piezoelectric effect under external mechanical energy but also have a wide light absorption range irradiated by visible light [201, 202]. In the past, TiO_2_/ZnO heterojunctions have been a common photocatalyst that can significantly promote interfacial charge transfer and separation. However, the piezoelectric properties of ZnO are usually ignored. Wang and coworkers adequately utilized the piezoelectric properties and light response characteristics of ZnO to design TiO_2_/ZnO nanowire arrays on stainless steel meshes. By introducing mechanical energy into the TiO_2_/ZnO nanowire arrays, the hydrogen production rate was greatly increased. The nanowire arrays also exhibited remarkable recyclability and stability due to the flexible stainless steel meshes. The excellent hydrogen production performance of the TiO_2_/ZnO nanowire arrays can be attributed to the piezoelectric field caused by the ZnO nanowires and the built‐in electric field, as shown in Figure 10L. The generation of the piezoelectric field originates from the bending of the ZnO nanowires. Under the high‐frequency of ultrasonic vibration, water generated great amounts of bubbles. The collapse of bubbles contributed to a high local pressure (approximately 100 MPa) on the ZnO nanowires, thereby leading to the bending of ZnO nanowires and a piezoelectric field across the nanowire (Figure 10M). The built‐in electric field between ZnO and TiO_2_ was caused by a constructed type‐II heterojunction. Owing to the different work functions of ZnO and TiO_2_ (∼5.2 and ∼5.1 eV, respectively), a type‐II heterojunction with a direction from TiO_2_ to ZnO was formed. As a result, photogenerated electrons can easily migrate from the CB of ZnO to the CB of TiO_2_, and the opposite migration direction can occur on photogenerated holes, which transfer from the VB of TiO_2_ to the VB of ZnO. This effective charge separation can be further enhanced by forming a piezoelectric field. A piezoelectric potential of around 10 V provided enough driving force for the separation of the photogenerated electron–hole pairs [203].

To further enhance the piezoelectric potential of piezo‐photocatalysts, designing a unique nanostructure is a promising method. For instance, a 1D asymmetric architecture of Au‐ZnO piezo‐photocatalysts exhibited excellent charge carrier separation and photocatalytic performance for degrading organic pollutants. As illustrated in Figure 10N, the unique asymmetric 1D nanostructure of Au‐ZnO can effectively regulate the spatially directed separation and distribution of the electrons and holes along the 1D nanostructure, achieving a larger piezoelectric potential to separate electrons and holes [202]. Since the generation of piezoelectric potential on piezoelectric semiconductors originates from the easier deformation under ultrasonic vibration, a variety of piezoelectric materials were designed as nanorod and nanosheet‐like structures. CdS, one of the most promising materials for photocatalytic hydrogen production, has been prepared in a nanosheet structure for easier deformation. The synergistic effect of light energy and mechanical energy endowed excellent hydrogen production efficiency. Measured results showed that CdS nanosheets with the piezoelectric and photocatalytic effect had a hydrogen yield rate of 633 µL h^−1^, which was twice higher than the CdS nanosheets with individual lighting conditions [201]. Besides the special nanostructure that can form polarization electric fields to promote charge separation, constructing lattice strains in the photocatalyst was also a promising method. Wang and coworkers introduced lattice strain into a CdS photocatalyst by regulating the crystallinity of the material, leading to a polarization of the internal electric field. By controlling the solvothermal temperature, CdS with lattice strain exhibited excellent charge separation of photogenerated electron–hole pairs, thus resulting in enhanced photocatalytic CO evolution from CO_2_ [204].

Recently, a few papers reported carbon‐based photocatalysts with piezoelectric effects under ultrasonic vibration for water splitting reactions and environmental remediation. It provides a novel idea for developing highly efficient and stable piezo‐photocatalytic materials [205]. Metal‐organic frameworks (MOFs) [206] have been widely adopted in the fields of pollutant adsorption, catalysis, and energy storage [207, 208]. However, little attention is focused on the applications of MOFs in piezo‐photocatalysis. Zhang and coworkers applied two isostructural MOFs, i.e., UiO‐66‐NH_2_(Zr) and UiO‐66‐NH_2_(Hf), in hydrogen evolution. Interestingly, both the photocatalytic performance of UiO‐66‐NH_2_(Zr) and UiO‐66‐NH_2_(Hf) were completely identical under visible light illumination, while the hydrogen production activity of UiO‐66‐NH_2_(Hf) was obviously higher than that of UiO‐66‐NH_2_(Zr) upon further ultrasonic irradiation. This result can be attributed to the stronger piezoelectric effect of metal‐oxo clusters Hf^4+^ in UiO‐66‐NH_2_, which provided a larger piezoelectric potential for driving charge separation and migration in comparison to Zr^4+^ [209].

### 4.5 Microwave Energy

MW radiation, with a frequency ranging from 0.3 to 300 GHz, induces interaction of the dipole moment of polar molecules or molecular ionic aggregates in alternating magnetic fields, thereby causing a homogeneous and fast thermal reaction [210]. In comparison to traditional heating methods, microwave‐assisted hydrothermal synthesis has been considered a promising method for preparing high‐quality photocatalysts due to its rapid heating, lower reaction temperature, and shorter reaction time. To this day, we have successfully synthesized a variety of photocatalyst materials, including ZnO [211], TiO_2_ [212], and Bi_2_O_3_ [213], which exhibit outstanding photocatalytic performance in pollutant degradation, water purification, and hydrogen evolution. In addition to practical applications in preparing high‐quality nanomaterials, MW radiation can also improve the photocatalytic activity during a photocatalytic process. In general, the improved photocatalytic activities through MW radiation can be attributed to two mechanisms: (1) thermal effect and non‐thermal effect, and (2) induced generation of highly oxidative ·OH radicals.

**FIGURE 11:**
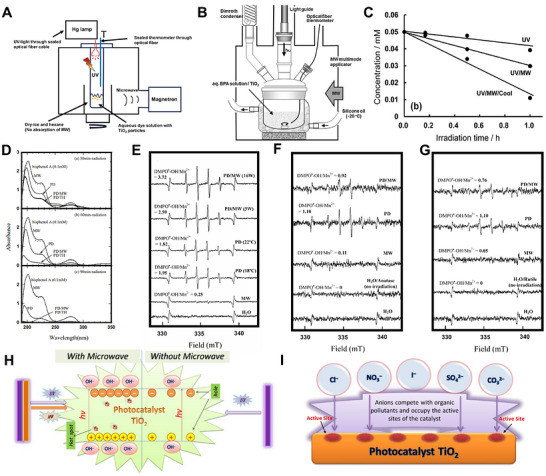
(A) Experimental setup used in microwave‐assisted photocatalytic degradation [214]. Copyright 2002, American Chemical Society. (B) Schematic diagram of the microwave‐assisted photoreactor (MPR) coupled with a cooling system. (C) Time‐dependent concentration curves of bisphenol‐A (BPA: 0.050 mM) by different reaction conditions: UV‐light photocatalysis (UV), microwave‐assisted photocatalysis (UV/MW), microwave‐assisted photocatalysis with a constant temperature (UV/MW/Cool) [216]. Copyright 2014, Elsevier. (D) Time‐dependent UV spectral patterns of bisphenol‐A (BPA) reflecting the cleavage of the aromatic ring(s) in BPA molecules by microwave degradation (MW), photocatalytic degradation (PD), microwave‐assisted photocatalytic degradation (PD/MW), and heating‐assisted photocatalytic degradation (PD/TH) routes [215]. Copyright 2002, Elsevier. (E‐G) Electron spin resonance (ESR) spectral features of the DMPO‐·OH spin adducts by UV light alone (PD route), the combined UV light and MW radiation (UV/MW route) for three various titania specimens: (E) mixed‐phase P‐25 TiO_2_. (F) pure TiO_2_ anatase. (G) pure TiO_2_ rutile [218]. Copyright 2003, Elsevier. (H) Schematic mechanism of restricted recombination of electron–hole pairs. (I) schematic illustration of the competitive adsorption of anions and environmental pollutants [219]. Copyright 2023, Multidisciplinary Digital Publishing Institute.

Horikoshi and coworkers first systematically investigated microwave‐assisted photodegradation using a classic photocatalyst, TiO_2_. The selected substrate was bisphenol‐A (BPA), which is an endocrine disruptor that tends to accumulate in nature with serious damage to animal species. The microwave‐assisted photodegradation apparatus is presented in Figure 11A [214]. It can be observed that the whole setup consists of a MW generator (2.45 GHz, 1.5 kW), a UV‐irradiation lamp (75 W), and a sealed thermometer. Researchers compared the photodegradation ability under four different external conditions: (a) the UV‐driven and microwave‐assisted photocatalytic degradation (PD/MW); (b) UV‐driven photocatalytic degradation (PD); (c) microwave‐assisted photocatalytic degradation MW; (d) the UV‐driven and thermal‐assisted photocatalytic degradation (PD/TH). UV absorption features of the two aromatic rings on BPA were applied to characterize the dynamics of the break‐up of the BPA phenyl rings. As shown in Figure 11D, the UV absorption peaks in the 195–300 nm spectral range vanished in the PD/MW and PD/TH routes, implying a complete photodegradation of BPA after a 90 min radiation. However, the other two routes, MW and PD, exhibit a higher peak intensity compared to their counterparts, indicating a weaker photodegradation of BPA. Since the photodegradation capability of PD/MW is similar to PD/TH, the improved photocatalytic performance may be attributed to the thermal effect originating from MW radiation [215].

To further reveal the influence of thermal effects and non‐thermal effects induced by microwaves on the photocatalytic performance, Horikoshi et al. designed a new microwave‐assisted photocatalysis setup for degrading BPA after ten years. Figure 11B shows an integrated microwave/photoreactor (MPR) system equipped with a cooling system. The incorporation of a cooling apparatus into the microwave‐assisted photocatalytic setup effectively excludes the effect of non‐thermal effects on photocatalytic activities. Different from previous research results, the photodegradation under a microwave‐assisted/external heating environment exhibited an excellent photocatalytic performance; the degradation process of BPA under controlled ambient conditions (UV/MW/Cool) with a constant temperature (21°C) shows a more degradation kinetics (6.5 × 10^−4^ mM min^−1^) compared to its counterpart under UV/MW conditions (3.3 × 10^−4^ mM min^−1^), as shown in Figure 11C. The above‐mentioned results reveal that the improvement of photodegradation of BPA not only derives from an MW thermal effect but also from remarkable non‐thermal effects. In this process, microwaves can cause rapid polarization or excitation of molecules, leading to increased reactivity or more efficient degradation, even at lower temperatures [216].

From the perspective of chemical reactions, an enhanced photodegradation in the MW/UV/TiO_2_ system is due to the generation of hydroxyl (·OH) radicals. Hydroxyl radicals, the second most reactive after the fluorine atom, play a crucial and highly beneficial role in photocatalysis, particularly in processes involving the degradation of organic pollutants, wastewater treatment, and energy production (such as hydrogen generation). Since ·OH has a redox potential of 2.8 V, it can attack a broad range of contaminants, including both hydrophobic and hydrophilic substances. In addition, the formation of ·OH can prevent the recombination of photogenerated electron–hole pairs, leading to an outstanding photocatalytic performance. In a TiO_2_ photodegradation system, the degradation process of organic pollutants can be achieved via the generation of ·OH on the TiO_2_ surface upon UV and MW radiation according to the following equation [217]

(6) $$\mathrm{Ti}O_{2}+\mathrm{hv}\to e^{-}+h^{+}$$

(7) $$h^{+}+H_{2}O\to \;\cdot \mathrm{OH}+H^{+}$$

(8) $$h^{+}+OH^{-}\to \;\cdot \mathrm{OH}$$

(9) $$e^{-}+O_{2}\to O_{2}^{-}\cdot$$

(10) $$O_{2}^{-}\cdot +H^{+}\to \mathrm{OOH}\cdot$$

(11) $$2\mathrm{OOH}\cdot \to O_{2}+H_{2}O_{2}$$

(12) $$H_{2}O_{2}+O_{2}^{-}\cdot \;\to \;\cdot \mathrm{OH}+OH^{-}+O_{2}$$

(13) $$\mathrm{OCs}+\cdot \mathrm{OH}\to CO_{2}+H_{2}O+\mathrm{salts}$$

Generally, the organic contaminants (OCs) are eventually decomposed into carbon dioxide, water, and salts by using ·OH as a strong oxidizing agent. Although the TiO_2_ photocatalyst under UV radiation can produce some ·OH to degrade organic pollutants, microwave‐assisted photodegradation can generate greater amounts of ·OH, which is attributed to a higher electronic excited state upon the MW radiation. Electron spin resonance (ESR) technology was applied to probe the formation of ·OH radicals in aqueous titania dispersions by using a novel device, where a MW generator was connected to both an ESR cavity and a photoreactor. At the same time, 5,5‐Dimethyl‐1‐pyrroline N‐oxide (DWPO) is a free radical scavenger to detect the presence of OH· radicals. Figure 11F–G clearly shows that the microwave‐assisted photodegradation technology can effectively increase the amounts of ·OH radicals by an ESR detection technology. Three different photocatalysts, mixed‐phase P‐25 TiO_2_, pure TiO_2_ anatase, and pure TiO_2_ rutile, were used to oxidize water molecules and organic pollutants under microwave irradiation (MW), UV irradiation (PD), and microwave‐assisted photodegradation (PD/MW). It can be observed that no evidently classic 1:2:2:1 spectral signature of spin‐trapped ·OH radicals in aqueous solution is detected. However, with the addition of different kinds of photocatalysts, the 1:2:2:1 spectral signature of the DMPO‐·OH spin adducts can be detected under PD and PD/MW conditions, implying an excited ·OH radical. Moreover, signal and intensity ratios of the DMPO‐·OH against the internal Mn^2+^ standard marker of PD/MW (16 W) are significantly higher than PD/MW (3 W), indicating that enhanced input power of MW can efficiently increase the amounts of ·OH radicals (Figure 11E). Interestingly, the increased temperature under UV irradiation from 18 to 22°C in Figure 11E has a negligible effect on the characterized signal of DMPO‐·OH spin adducts, illustrating thermal effects caused by MW radiation cannot contribute to the increase in ·OH radicals. Furthermore, authors compared signal radios of DMPO‐·OH/Mn^2+^ in different types of photocatalysts. It can be found that the P‐25 TiO_2_ exhibits a stronger signal radio of DMPO‐·OH/Mn^2+^ in comparison to pure TiO_2_ anatase (Figure 11F) and pure TiO_2_ rutile (Figure 11G), implying an excellent photocatalytic degradation [218].

Microwave‐assisted photocatalytic degradation on TiO_2_‐based materials is an advanced oxidation technology based on using ·OH radicals to degrade environmental pollutants. Previous research has confirmed that the introduction of MW can produce more ·OH compared to a pure photocatalytic system. The increased amounts of ·OH are mainly ascribed to two mechanisms: (1) Enhanced molecular vibration and dipole rotation; (2) Improved activation of photocatalysts. Firstly, microwaves interact with polar molecules (e.g., water) and materials with dipoles, inducing rapid oscillation and vibration. This leads to localized heating and creates “hot spots”, increasing the possibility of breaking chemical bonds to generate reactive species such as hydroxyl radicals (Figure 11H). Secondly, the incorporation of MW into the TiO_2_ degradation process probably creates additional defect sites, which leads to an improved charge separation and restricted recombination of electrons and holes. Thus, an improved activation of photocatalysts can be obtained [219].

Additionally, enhanced surface hydrophobicity under irradiation of both MW and UV‐light may further increase the amounts of ·OH radicals [219]. It is worth noting that untreated pollutants probably have a variety of anions, such as Cl^−^, CO_3_^2−^, SO_4_^2−^, NO_3_^−^, I^−^, etc. These anions can be adsorbed on the surface of photocatalysts, occupying the active sites, leading to a reduced efficiency of photocatalytic degradation (Figure 11I). On the other hand, these anions can react with ·OH radicals, further consuming a certain amount of ·OH radicals that participate in pollutant degradation. For instance, the presence of CO_3_^2^ can consume ·OH radicals and change the pH of the system, causing a negative effect on the photocatalytic efficiency and stability [220]. NO_3_^−^ can react with water molecules under irradiation of solar light to generate some ·OH radicals. It counteracts the side effects of occupied active sites on the photocatalytic performance to some extent [221].

Recently, microwave‐assisted photocatalysis has made new progress in degrading tetracycline (TC) with ZnO photocatalysts. It can be found that the degradation efficiency of TC under microwave‐assisted photocatalysis is 4.3 times higher than its counterpart without the assistance of microwave. The improved photocatalytic performance can be attributed to the enhanced light absorption and charge separation efficiency. The UV–Vis DRS showed that the light absorption edge of ZnO shifted from 390 to 450 nm after a microwave‐assisted photocatalytic reaction. In addition, Semi‐in situ photochemical tests demonstrated that MW can induce more photogenerated electron–hole pairs and hydroxyl radicals generated during photocatalysis, leading to an enhanced photocatalytic performance [222]. Microwave‐assisted photocatalysis has been studied for at least 20 years, but the deeper mechanisms of how microwaves affect light absorption, charge separation, surface chemical reaction, and surface mass transfer are not clear. Future research in microwave‐assisted photocatalysis should focus on building a mutual relationship between MW effects and chemical reaction kinetics and designing a suitable MW transmitting device that can be utilized in the large‐scale photocatalytic setup.

## 5 Conclusions and Future Prospects

In conclusion, photocatalysis based on solar energy conversion is one of the most promising technologies for addressing energy crises and climate change. The current research hotspots focus on increasing the photocatalytic efficiency in three crucial steps, including the light absorption efficiency, the charge separation efficiency, and the surface reaction kinetics. Conventional addressing strategies are composed of element doping, defect, and heterojunction construction, bandgap engineering, and facet engineering. However, the above‐mentioned methods generally introduce additional defects or recombination, leading to reduced efficiencies. The introduction of other energy, such as thermal energy, electrical energy, magnetic energy, mechanical energy, and MW energy, not only can efficiently avoid the damage to the semiconductor itself, but also provide a strong, stable, and widely distributed driving force to improve charge separation and transfer. In this review, we systematically summarize fundamental mechanisms of five energy fields and the advantages of the external energy‐coupled photocatalytic processes. In particular, we comprehensively review the recent developments of novel materials preparation that are sensitive to external energy input and new multi‐energy integrated photocatalytic device designs. Moreover, the practical application directions, including water splitting, CO_2_ reduction, valuable chemicals synthesis, and pollutant degradation, are also involved.

Although considerable achievements have been made in multi‐energy‐coupled photocatalytic processes, some emerging challenges should not be ignored.

1. Currently, a few semiconductor materials are sensitive to the external energy‐field except for solar light. Therefore, developing more polarization materials (piezoelectric, pyroelectric, and ferroelectric materials) and investigating fundamental polarization mechanisms is crucial and necessary [102]. A variety of polarization materials are non‐centrosymmetric (NCS) materials with a significant polarity. The polarity of NCS materials can be attributed to the non‐centrosymmetric arrangement of either ions or ionic groups (non‐overlap of the positive and negative charge centers), thus inducing positive charges on one side and negative charges on the other side, forming an electric polarization field from one region to another region [223]. For photocatalytic polarization materials, the polarization electric field plays a vital role in improving the charge separation efficiency on the bulk and surface compared to traditional non‐polarized semiconductors, which is due to the generated strong polarization electric field caused by their non‐centrosymmetric structures.
2. The coupling of multi‐energy fields possibly causes synergistic effects for improving the photocatalytic performance. In the process of photocatalytic CO_2_ reduction, using a photo‐thermal‐magnetic coupling field on a metal‐organic‐framework‐based monolithic NF@ZnO/Au@ZIF‐8 photocatalyst can significantly elevate the temperature to about 180°C, leading to an improved charge transfer and increased photocatalytic efficiency [224]. However, in these complex photocatalytic systems with multi‐energy fields, distinguishing every influence of a single energy field on charge carrier behavior is difficult, which restrains in‐depth understanding for multi‐energy integrated photocatalysis [225]. Therefore, designing rational comparative experiments to clearly investigate the influence mechanisms of every external energy field is indispensable.
3. Valuable chemical synthesis by solar energy conversion, such as glycerol oxidation, 5‐hydroxymethylfurfural oxidation, and artificial nitrogen fixation, has attracted a large amount of attention due to the tremendous potential for green chemical synthesis [226, 227]. At present, external‐energy‐assisted photocatalytic processes have demonstrated excellent selectivity for specific synthetic reactions in comparison to single‐mode photocatalysis, but most research just focuses on the improvement of selectivity and production instead of in‐depth mechanisms on a microscale due to the lack of effective research methods. With the development of in situ spectroscopy technology in recent years, organic synthesis by electrocatalysis has achieved considerable progress. Some in‐depth understanding of the reaction selectivity on a microscale has been revealed. If we can apply the new in situ spectroscopy technology to multi‐energy‐coupling photocatalytic chemical synthesis, it will open up a new perspective to understand deep reaction mechanisms.
4. Until now, a variety of research about multi‐energy integrated photocatalysis has been limited in the laboratory, with only a few studies concentrated on practical applications. In fact, the current solar energy conversion efficiency in water splitting has reached commercial standards. Although the price of “green” hydrogen is still higher than that of “gray hydrogen” produced by industrialized steam methane reforming (SMR) [228], the future price advantages of “green hydrogen” will become more competitive compared to “gray hydrogen” due to the gradually increasing carbon emission price. Therefore, designing large‐scale, high‐efficiency, and stable photocatalytic devices will become a research hotspot in the future. The primary work should focus on the device lifetime. For bias‐free PEC setups, the designing of large‐scale reactors, selection of suitable electrolyte and ion exchange membranes, distribution of photovoltaic voltages, and removal of surface bubbles are key factors that need to be considered, which would provide new research directions for scientists.

## Conflicts of Interest

The authors declare no conflicts of interest.
